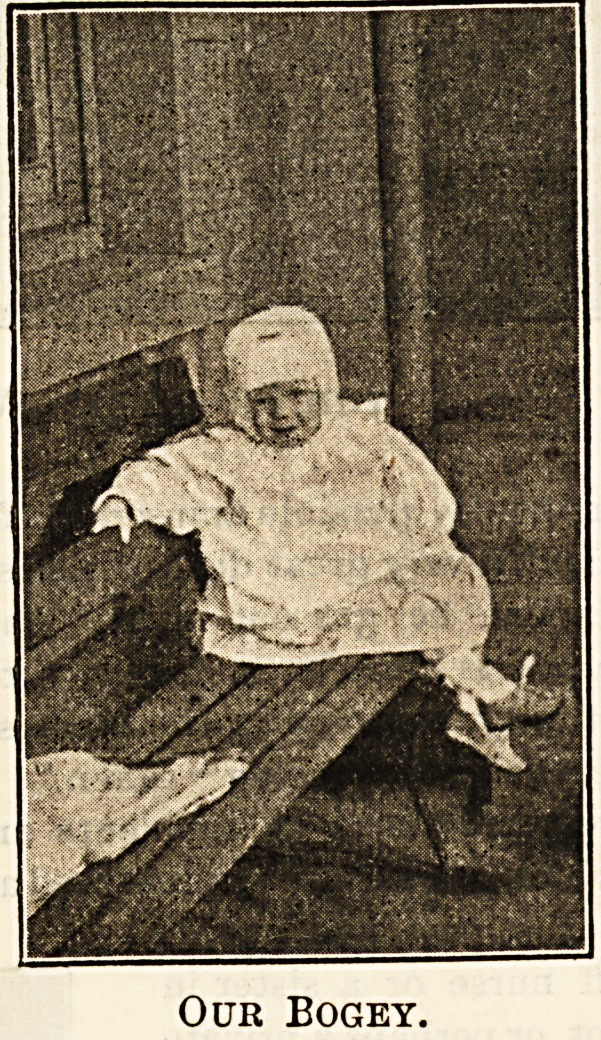# The Hospital. Nursing Section

**Published:** 1902-10-04

**Authors:** 


### The Hospital.

### Nursing Section.

Contributions for this Section of "The Hospital" should be addressed to the Editor, "The Hospital" Nursing Section, 28 & 29 Southampton Street, Strand, London, W.C.

No. 836.—Vol. XXXIII. *SATURDAY, OCTOBER 4, 1902.*

### Notes on News from the Nursing World.

### THE SOUTH AFRICAN WAR MEDALS FOR NURSES.

It is announced, in a special Army Order dated September 29th, that the new war medal in silver, bearing the portrait of King Edward, is to be issued to all nursing sisters, as defined in Army Order 195, of 1901, provided that they were actually serving in South Africa on or after July 1st, 1902, and that on that date they had completed 18 months' war-service, or subsequently completed such service before June 1st, 1902. The ribbon will be orange, white, and green, in three stripes of equal width, and so worn that the green stripe shall be on the right. Claims for medals should be addressed to the Director-General, Army Medical Service, War Office, S.W.

### THE WOMEN'S MEMORIAL TO QUEEN VICTORIA.

It has been decided that the fund now being raised by the women of England on behalf of the Queen Victoria Jubilee Institute for Nurses shall be closed at the end of the year. To keep a fund open for an indefinite period is always a mistake, and the committee, in arriving at their determination, have given ample notice to the organisations at work in the country on behalf of the movement. At present £60,000 of the £70,000 required is in hand or promised, and there should not be much difficulty in raising the balance before Christmas. Special exertions are being made just now in Birmingham and Staffordshire, and if similar efforts should, as we hope, become general, the result can hardly fail to be satisfactory. But there must be no slackening of effort so long as the issue is in doubt. Every contribution that can be collected, from a penny upwards, will be needed to bring the total up to the amount which the women of England gave to Queen Victoria at the Jubilee in 1887—an amount that she characteristically used for the purpose of founding the beneficent organisation that bears her illustrious name.

### THE ROYAL RED CROSS.

It is announced that the King has conferred the decoration of the Royal Red Cross on Miss Annie Myers and Miss Daisy Brazier in recognition of their services in attending to the sick and wounded soldiers in Peking during the recent operations in China. To prevent misunderstanding, we may state that these ladies are not nurses but were connected with one of the legations in Peking. They are mentioned in the report of the British Minister in China respecting events in Peking, 1900, as follows:—"Especially to be commended are two young ladies (Miss Annie Myers and Miss Daisy Brazier) who boiled and filtered the water for the hospital—a by no means easy task in the tropical heat, with a hand-pump filter—and carried it as often as not with the bullets whistling and shells bursting round them. Miss Brazier, while on her errand of mercy, was struck by the fragments of a shell, but, happily, only slightly. Dr. Poole in his report will, I am sure, bear testimony to the devoted behaviour of the hospital nurses and ladies who assisted them in their work." It cannot be said that there has been any undue haste in acknowledging the devotion and courage of the two ladies who now, after the lapse of two years, have been awarded the decoration of the Royal Red Cross.

### QUEEN ALEXANDRA'S IMPERIAL MILITARY NURSING SERVICE.

We understand that the appointment of Miss Sidney Browne as matron-in-chief of Queen Alexandra's Imperial Military Nursing Service, which was announced as temporary, is likely to be made permanent, and that Miss Browne will thus become the acknowledged head of the service. The vacancy on the nursing board of the service has, we are officially informed, been filled by the selection of Miss Mabel Cave, one of the youngest and most able of London matrons. Miss Cave, who succeeds Miss Gordon, the late matron of St. Thomas's Hospital, in this capacity, was trained at the London Hospital, where she was afterwards sister. In 1897 she was appointed matron of the Metropolitan Hospital, and in 1898 matron of Westminster Hospital. We heartily congratulate Miss Cave upon the honour conferred upon her, and we rejoice to learn that her new duties will not prevent her from continuing one of the nurses' representatives on the Council of the Royal National Fund for Nurses, and a Member of the Committee of the Junius S. Morgan Benevolent Fund.

### THE LATE LADY SUPERINTENDENT AT NETLEY.

On Wednesday Miss Norman, whose resignation was sent in to the War Office some little time since, left the Royal Victoria Hospital, Netley. In consequence of ill-health she was granted six months' leave in the early part of the year, and returned to duty on August 1st, but speedily found that she was not strong enough to continue to discharge the duties of lady superintendent. Her departure will be much felt by the patients with whom, though having no actual nursing to do among them, she always kept in touch. It was her custom to visit the wards during some part of the day and talk to the men individually; in fact, she took a personal interest in them all, and her kindness of heart and sympathy of manner made her a universal favourite. The sisters who have worked with her at Netley for the last two and a half years of the war presented Miss Norman with a travelling tea basket as a small token of their great esteem.

### THE VICEROY OF IRELAND AT THE MEATH HOSPITAL.

On Thursday last week, the day after their arrival in Ireland, the new Lord-Lieutenant and the Countess of Dudley paid a visit to the Meath Hospital in Dublin. They were received by the members of the medical and surgical staff and were conducted through the wards by the lady superintendent, Miss Ellinor Lyons, and Dr. Russell Magee, house surgeon. They appeared much interested not only in the hospital but in the patients, to each of whom they spoke some cheering words, especially to one case, a young man who had met with a terrible accident which necessitated the amputation of both legs and one arm, but who is now able to go about his ward in a wheeled chair. They spent about two hours inspecting the hospital, and before leaving Lord Dudley made the following entry in the visitors' book :—"My first visit to any institution in Dublin, and I am delighted with it." Lord and Lady Dudley expressed themselves particularly pleased with the appearance of the sisters and nurses, who were grouped on either side of the steps in their pretty uniforms of white, blue, and grey. The nursing at the Meath Hospital is done by the Dublin Red Cross Sisters, of whom the lady superintendent, Miss Ellinor Lyons, is the head. This institution was founded in 1885, and sends all its probationers to be trained in the Meath Hospital. A considerable number of the Meath nurses have obtained excellent posts in England and abroad, and several, on the outbreak of the war, joined the Army Nursing Reserve, and were sent to South Africa. On the day after the viceregal visit Lady Dudley drove to the hospital in her motor, bringing flowers and books for one of the patients in whom she was specially interested.

### DENGUE FEVER AT THE CIVIL HOSPITAL, HONG KONG.

The latest scourge afflicting Hong Kong is dengue fever. The hospitals in the island have overflowed with patients of all ages and descriptions, and those not admitted for that disease have soon contracted it. Among the staff at the hospitals the first to be attacked were the ward boys and native nurses, and in the last week of August seven of the sisters of the Government Civil Hospital were off duty with it, some in the initial stage, others convalescing. The invalids rendered *hors de combat* by an inconvenient, rather than dangerous, disease are Sisters Shelbourne, Renwick, Watson, Stollard, Gorham, Millington, and Barr.

### THE POSITION IN JERSEY.

Trained nursing has yet to win its victories in Jersey. From the account which our Commissioner gives of an inquiry on the spot into the position, it would seem that at the institution which should be a centre of light and leading in the matter, there is a strong prejudice in favour of untrained nurses. At the hospital in St. Helier they are neither employed nor desired. The handy girl who can satisfy the doctor that she is physically sound is mistress of the situation ; even the recovered patient who is willing to be converted into a nurse by donning a suitable attire is preferred to the owner of a three-years' certificate. Under the auspices of an untrained matron, there is no division of day and night duty, night nurses are unknown, and the untrained young woman, or young man, who is styled surgical or medical nurse, makes the ward a sitting-room in the day and sleeps in it at night. The position is a little better at the General Dispensary. But the fully-trained nurse in command of the eight beds in the little infirmary, has only an untrained assistant ; there was once a district nurse attached to the dispensary, but she had to be sent away because there was not enough money to pay her salary. There are apparently two or three trained private nurses on the island, but two nursing homes have ceased to exist, partly because of the alleged "flightiness" of some of the staff. Perhaps it is a fear of that flightiness which has closed the doors of the hospital against the trained nurse ; and possibly the French sisters have earned their well-deserved reputation partly owing to their freedom from flightiness. Outside the gates of the hospital, it is still thought, however, that trained nurses might have a chance, if the right sort were imported. We have not the least doubt of it, but as long as the residents are satisfied that the poor should be entirely tended by untrained male and female attendants, excellent and devoted as they may be, we are afraid that so far as trained nursing is concerned, Jersey will remain behind the times.

### A NEW HOME AT STOKE-UPON-TRENT.

There has just been opened at Stoke-upon-Trent, in connection with the Spittals Workhouse Infirmary, a most comfortable and beautifully furnished nurses' home, with a fine red-ash tennis-court attached. It is planned to accommodate thirty nurses, and contains on the ground floor various sitting-rooms, dining-room, writing-room, kitchens, and store-rooms, while the first and second floors are mainly occupied by bedrooms. The cost, including electric light installation, but not furnishing, is nearly £4,000. At the opening ceremony, which was happily performed by the chairman of the Stoke-upon-Trent Board of Guardians, some very pleasant things were said about the nurses, and an interesting feature of the proceedings was the presentation of certificates to eight probationers—namely, Misses Butler, Yates, Scott, Daniel, Gibson, Spruce, Marfleet, and Durber. We are glad to learn that in this case the relations existing between the nursing staff and the master and matron of the workhouse are of the most cordial and satisfactory character.

### PRIVATE NURSES AND SMALL-POX CASES.

A claim was made last week by Bessie Alice Bowater Harrington, proprietress of the North London Nursing Institute, Alexandra Road, Finsbury Park, against the Tottenham Urban District Council for £43 1s. for services rendered under a contract made between plaintiff and defendants. It appears that on January 28th the Council's inspector asked plaintiff if she would supply nurses during the small-pox epidemic. The plaintiff said that she would put the matter before her nurses, but if they took up the work, it must be purely voluntary. The nurses expressed themselves willing, so long as an arrangement was made by her with the Council that each nurse was to receive £3 3s. a week. Four nurses were sent, each one filling up a form, which stated that 24 hours' notice on the part of the person making the engagement had to be given in order to terminate it, or in lieu one week's wages to be paid the nurse on leaving. Nurse Moules—who was in receipt of only £18 a year, with uniform, from plaintiff—went to the hospital, found the work very heavy, and wrote to plaintiff pointing out that she was risking her life, and asking for a commission. Plaintiff replied that that was all nonsense, and sent her a cheque for her month's wages. This Nurse Moules returned in place of notice, and left the plaintiff's service. She was subsequently taken ill and quitted the hospital for a time, her wages being paid her while away by the District Council, who engaged her as their nurse upon her return. Plaintiff claimed the £43 1s. from the Council for the whole 13 weeks, during which period they employed Nurse Moules, although the nurse had resigned service with plaintiff after six weeks. The verdict was for the defendants with costs, the judge holding that as Miss Moules returned the cheque to plaintiff instead of notice, and plaintiff accepted it, the contract had terminated.

### OBEDIENCE TO HOSPITAL RULES.

In the first of a short series of "Words of Advice to Nurses," in our issue of to-day, Miss Esther H. Young, whose appointment as Matron of the Howard de Walden Nurses' Home is just announced, enforces among other duties that of punctuality. Speaking with the authority of her experience as assistant matron at Addenbrooke's Hospital, Cambridge, and Matron at Guy's, she lays stress, too, upon a point which, we are afraid, is often lost sight of. Nurses who remain in their wards after their time are, she correctly says, just as disobedient as if they went on duty half an hour late. Miss Young rightly insists upon the importance of remembering that hospital hours are arranged by those in authority, and should not in any case be disregarded. With respect to uniform, she, of course, admits that there are differences of opinion, but here again she is on safe ground when she adds, "Obey the rule." In fact, the lesson of her article is the imperative duty of literal obedience to all regulations in force—a lesson which certainly requires to be brought home to some nurses.

### THE VINDICATION OF THE LYNN NURSING STAFF.

A member of the Lynn Board of Guardians sprung upon the meeting on September 5th a very serious charge against the members of the nursing staff. According to Mr. Kidd's statement, the nurses had withheld the allowances of brandy ordered by the medical officer for certain infirmary patients in order to use it for themselves. No answer was attempted at the time because no one was aware that the charges would be made, but the nurses at once took action to clear their reputation, and presented a letter to the Chairman of the Board, in which they requested him at once to obtain the removal of the stigma that had been cast upon their characters without cause. The letter was signed by the six nurses, M. Rogerson, M. Westwood, E. Robbins, K. Rozier, G. Vincent, and R. E. Chilvers. When the matter came before the Board the Chairman first read a letter from the medical officer dealing categorically with the charges of neglect to administer stimulants, and it then transpired that of the patients named three were dead and the other had left the infirmary. The medical officer was, however, able to state, from his own knowledge, that no complaints were ever made by two of the deceased, while a remark alleged to have been uttered to himself by the third was quite untrue. In respect to the fourth patient, she had volunteered a statement which was to the effect that she had brandy three times a day while she was in the infirmary and never missed a dose; also that she never told Mr. Kidd, or anyone else, that she had not received it. Having heard the Medical Officer's report, the Chairman added various reasons why he was convinced that there was no foundation for the charges, and the whole of the guardians, except Mr. Kidd, assented to a vote of confidence in the nursing staff. Miss Rogerson was present during the discussion, and at its conclusion the rest of the nurses were asked into the room, and personally received the assurance of the guardians that "they had done their duty in the distribution of brandy as well as their other duties." We congratulate the staff upon the result of an accusation of a most odious nature, which, had it not been completely refuted, would have proved them to be unfit for their work.

### LECTURES AT KING'S COLLEGE HOSPITAL.

The usual course of lectures to the nursing staff at King's College Hospital will begin in November, when all the holidays are over. They will comprise the following subjects :—Anatomy, Physiology, Surgical, Medical, and Obstetric Nursing. Hygiene, the Nursing of Diseases of the Eye, Diseases of the Ear, and Diseases of the Throat and the Nursing of Sick Children. Dr. John Curnow, whose death deprives the nurses of a valued friend, will be greatly missed during the winter course of lectures, in which he took for many years a prominent part. The courses will be delivered by Drs. Norman Dalton on Physiology and Medical Nursing, Albert Carless on Anatomy and Surgical Nursing, Raymond Crawfurd on Hygiene, L. Vernon Cargill on the Eye, Arthur H. Cheatle on the Ear, Macay Macdonald on the Throat, Hugh J. M. Playfair on Obstetric Nursing, and G. F. Still on the Nursing of Sick Children. The lectures will be given on Tuesdays and Fridays, and will continue until June. Classes in connection with the courses will be held by the home sister.

### NURSES FROM SOUTH AFRICA.

The following nurses have recently arrived at Southampton from South Africa :—In the *Galician*, sisters W. Smith, A. E. Ball, and E. J. Law; in the *Tagus*, sisters A. A. Murphy and A. Twamley; in the *Briton*, sisters A. Rose-Innes and A. Jarman; on the *Canada*, sisters M. Dow and V. Lamb; and on the *Guelph*, sisters C. Duncan, M. L. Tyndall, and L. Thornton.

### SHORT ITEMS.

There are rumours that the post of matron of one of the principal London hospitals will shortly become vacant.—Under the will of Mrs. Selina Lingham, of Vincent Lodge, Herne Hill, who died last August, the Mildmay Park Nursing Institution, and Princess Christian Nursing Home at Windsor, will benefit to the extent of £500 each.

### Lectures to Nurses on Anatomy.

By W. Johnson Smith, F.R.C.S., Principal Medical Officer, Seamen's Hospital, Greenwich.

LECTURE XXVII.—THE ORGANS OF THE NERVOUS SYSTEM.—(*Continued from page* 333.)

*Base of Brain.*—The base or lower surface of the brain, from its complex conformation and the number of nerves attached to it, presents a strong contrast to the even and simple upper surface. It should be studied in connection with the interior of the base of the skull, which, as was pointed out in Lecture VII., presents on either side, from before backward, three well-marked divisions—the anterior, middle, and posterior depressions, or fossæ, of the cranium. On the level surface of the anterior fossa, which forms the roof of the eye socket, rests the anterior portion of the cerebrum, formerly called the anterior, and now the frontal, lobe. Behind this, resting on the deep middle fossa, we see the middle, or, as it is now called, the temporal lobe. The deep cleft between the frontal and temporal lobes is formed

Fig. 61.—Base of the Brain.

A, longitudinal fissure; B, corpus callosum; C, optic chiasma; D, pituitary body; E, right crus cerebri; F, pons Varolii; G, bulb, or medulla oblongata; H, cerebellum.

The figures from 1 to 12 indicate the corresponding cranial nerves.

by part of the fissure of Sylvius, which we have already noticed, on the outer surface of each cerebral hemisphere. In the portion of brain corresponding to the still deeper posterior fossa we lose sight of the proper brain, or cerebrum, and find that this depression is almost wholly occupied by the corresponding lobe of the cerebellum, or little brain.

In the middle line of the base of the brain from the front to the back we notice (fig. 61, A) part of the long fissure which divides the cerebrum into the right and left hemispheres; behind this (B) a part of the intervening band of nerve tissue known as the *corpus calosum*, next (C) the *optic commisure* or *chiasma* (C) formed by the meeting of four large nerves, two in front—the *optic nerves*, diverging each to the corresponding eyeball, the two behind, the *optic tracts*, which pass for some distance backwards and outwards and enter the brain. Behind the optic commissure is a small rounded body—the *pituitary body* (D)—composed partly of gland tissue, partly of nerve cells, attached to the surface of the brain by a thin stalk called the *infundibulum*; this small and apparently insignificant structure must exercise or take a part in some function at present quite unknown, as it is often found enlarged and diseased in fatal cases of the interesting disease known as acromegaly, in which enlargement of the bones of the face and of the hands and feet is a characteristic feature. Behind the pituitary body bounding, together with the diverging optic tracts, a diamond-shaped space, are the two thick columns of white nerve tissue, known as the *crura* or *peduncles of the cerebrum* (E). We come next to the bridge of Varolius—the *pons Varolii* (F), which forms a conspicuous feature on the under surface of the brain, and to the thickened upper end of the spinal cord—the *bulb* or *medulla oblongata* (G) resting on the cerebellum. Quite at the back of this surface of the brain we notice the posterior part of the cleft between the two cerebral hemispheres, and also a shallow notch in the little brain indicating a partial division of this organ into two lateral hemispheres. The little brain, it will be seen, does not present tortuous convolutions like those on the surface of the cerebrum, but is traversed by regularly disposed slits or furrows, which take a curved course from side to side across each hemisphere.

The base of the skull, as was pointed out to you in the seventh lecture, is perforated by numerous holes or *foramina* for the transmission of nerves and blood-vessels. The nerves which pass from the cranium to external organs and are called *cranial nerves*, are arranged in twelve pairs, the dozen on one side corresponding in every respect to the dozen on the other side of the brain. The nerves followed in order on one side of the base of the brain from before backwards are (fig. 61):—

1. *The Olfactory Nerve* or nerve of smell (1), a long rod of grey matter with a swollen end, placed on the anterior lobe of the cerebrum near the fissure between the hemispheres. Really it is not in the usual sense of the word a nerve at all but a small outlying portion of brain from the under surface of which numerous very minute nerves pass through orifices in the bony roof of the nose.

2. *The Optic Nerve* or nerve of sight (2) which, as has already been stated, is derived from the optic commissure, passes as a single firm and round nerve through a round hole at the apex of the eye-socket and is inserted into the back of the eye-ball. At the commissure some of the nerve-fibres pass over to the optic nerve on the opposite side, others to the optic tract behind the commissure on the same side, whilst others intersecting with those from the other eye, pass to the opposite optic tract.

The perfect correspondence of the movements of the eye-balls and the perception of a single instead of double visual images depend partly on the arrangement of nerve-fibres in the commissure, and partly upon the proper and harmonious action of the small but important muscles which move the eye in different directions. Of the four straight muscles or *recti* which move the front of the globe inwards, outwards, upwards, and downwards, three are supplied by the

### LECTURES TO NURSES ON ANATOMY.—*Continued.*

third cranial nerve (3) and one, the external rectus which moves the pupil outwards, by the sixth (6) cranial nerve. Of two other small muscles, the *superior and the inferior oblique*, which take part in other and more complex movements of the globe, the latter is supplied by the third nerve, whilst the former is exclusively supplied by the fourth nerve (4).

The fifth (5) called the *trigeminal*, the largest of the cranial nerves, has an extensive distribution and a varied function. It gives off a nerve of special sensation, that of taste, motor branches to the muscles which move the lower jaw and take part in mastication, and widely diffused sensory branches to the head and face. These sensory branches are the offending structures in the ordinary forms of headache and in neuralgia. Unlike the other cranial nerves, and, as we shall learn in a future lecture, like nerves derived from the spinal cord, it has two roots at its origin from the surface of the brain, a small and simple root containing only the motor nerve-fibres, and a larger and exclusively sensory root swollen by a large mass of grey matter, called the Gasserian Ganglion.

The numerous minute muscles of the face, which play so important a part in the expression of emotions, are supplied by the seventh nerve (7).

The eighth, or *auditory nerve*, is the special nerve of hearing (8).

The ninth nerve (9), called the *glosso-pharyngeal*, is another mixed nerve. It is supplied to the back of the tongue, where it takes a part in the special sense of taste, and also to the muscles of swallowing.

The tenth nerve (10), though not the largest of the cranial nerves, has the most extended distribution, as it supplies branches—some motor, others sensory—to important organs in the neck, chest, and abdomen. It is usually called the *pneumogastric*, from its relation to the organs of respiration and to the stomach. From its length and extent it was termed by the old anatomists the *par vagum* and *vagus*. Amongst the many important structures to which this ninth nerve is distributed may be included the organs of voice, the muscles of swallowing, the air passages and the lungs, the heart, and the stomach. Sir Charles Bell says: "The recollection of this distribution will explain to us many sympathies; for example, the irritability of the larynx in exciting coughing; the hysterical affection of the throat when the stomach is distended with flatus; the exciting of vomiting by tickling the throat; the effect which vomiting has in diminishing the sense of suffocation; the relations betwixt the heart and lungs, the lungs and stomach, and the stomach and heart."

The eleventh nerve (11)—the *spinal accessory*—has a double origin, some of its nerve fibres derived from the brain, being associated with the pneumo-gastric, whilst the major part derived from the cervical portion of the spinal cord, acts as the motor nerve of the two large muscles—sterno-mastoid and trapezius—by which the head is nodded and turned from side to side.

The twelfth and last cranial nerve, called the *hypo-glossal* (12), is the motor nerve to the muscles of the tongue.

Of the twelve pairs of cranial nerves, three (1st, 2nd and 8th) are wholly nerves of special sense; five (3rd, 4th, 6th, 7th and 12th) are wholly motor nerves, and the remaining four (5th, 9th, 10th and 11th) mixed nerves of motion and sensation, and in two instances (5th and 6th) also of special sense.

Most of these nerves have their origin at or near the bulb or medulla oblongata, which by reason of the physiological importance of the organs to which these nerves, particularly the pneumogastric, are distributed, may be regarded as the most vital part of the body.

### Nursing in the Island of Jersey.

BY OUR COMMISSIONER.

When I determined to take advantage of a visit to Jersey to ascertain the position of nursing in the island, I naturally made my way, first of all, to the imposing and extensive establishment in St. Helier, which serves the purpose of a general hospital and poor-house. My reception was of an extremely courteous character, and when I had explained my object, the Director not only afforded me every facility, but in company with the head nurse, the matron being absent, showed me over the whole of the place, including even the padded rooms in which violent persons are temporarily confined. The hospital, which was opened in 1862, and was erected on the site of a building twice destroyed by fire, does credit to those who planned it. Not only the sick wards, which are quite distinct from the poor-house, but the whole of the rooms, are large and lofty, the corridors are spacious and airy, and the use of Jersey granite gives distinction, as well as solidity, to the general appearance of the interior. The main staircases are of iron. The charming show of flowers in the garden imparts a cheerfulness to the exterior which is reflected by most of the inmates, for whom, so far as material comfort is concerned, everything possible under the circumstances appears to be done.

### No Trained Nurses.

But there are no trained nurses in the hospital. And there is not, according to the Director, a desire that any should be introduced. The matron, who is the wife of the Director, and was previously the "surgical nurse," has only enjoyed the same experience as that of most of the members of the staff. This experience is regarded in the hospital as a practical training, and nothing more, it is contended, is necessary. On the female side there are usually in the medical and surgical wards about fifty patients, and these are tended by four nurses, there being also a nurse for the maternity ward, and one who looks after the aged and infirm in the poor-house. The nurse for the maternity ward has recently been added.

### Male Assistants.

The male patients are nursed entirely by males, five in number, the wards being of the same size as the wards on the female side, but the average number of patients rather larger, owing to the greater frequency of accidents to men. This has always been the custom, and the head male nurse has been in the establishment about 20 years. His mother was head nurse on the female side for 18 years. In all there are a hundred beds in the infirmary, while in the poor-house there are 225 always ready. Another hundred could, I was informed, be rigged up at a pinch. The male and female nurses are liable to be sent to the fever hospital at West Mount, which has 30 beds, or the small-pox hospital at Overdale, which has 36. Both are within easy distance, but there has not been a case of small-pox for more than two years.

### Age of Admission.

Nurses of either sex are admitted at 18, and, though there is no age limit, preference is given to persons

### NURSING IN THE ISLAND OF JERSEY—*Continued.*

between 18 and 22. The last female nurse appointed was taken from among the patients after she had recovered. But there is no difficulty in obtaining nurses, and the only examination to be faced is that of the doctor, who gives a certificate of physical fitness, and the applicant is engaged forthwith, usually but not necessarily after a month on trial, as a medical nurse at a salary of £16. This is increased to £20 when the nurse is transferred to the surgical ward. Applications are often received from England, but Jersey applicants always have the preference, partly because it is absolutely essential that the nurses should be able to speak both English and French fluently. There are many French labourers who do not understand a word of English, and I understand that scarcely one out of ten applicants for admission from England can fulfil the condition as to language.

### No Night Nurse.

Each nurse has a ward allotted to her care—the same rule being observed on the male side—and she is on duty day and night, there being no night nurse in the entire establishment. The nurse has no private room, and not only sits in the ward if there are no bad cases, but sleeps in it always. There is an operation room on each side, and there are frequently serious operations, which are attended by the surgical nurses. The doctor, who is not resident, is master in the infirmary, and the director in the poor-house. Cases of diphtheria are sometimes treated in the medical wards, and at other times are isolated. The nurses are allowed to be off duty two evenings during the week, and every other Sunday from two to ten; a fortnight's holiday is given. It is not a rare event for a nurse who has been at Jersey three or four years to enter a London hospital for training, and one who was there a short time ago, is now a district nurse in Manchester. The patients are of all nationalities, notably Norwegians, Dutch, and Germans, and many of them being sailors, there are strange and interesting cases.

### Paying Patients.

Although the hospital is supported by the State, and is governed by a board consisting of three jurists, three rectors, twelve constables, or mayors, and three deputies, the inmates who can afford to pay, whether in the hospital or in the poor-house, contribute half a guinea a week for their support. Any person, whether a native of Jersey or not, who obtains a permit from a member of the Board, is received as an inmate. If a native, the cost is charged to his parish; if not, it is divided among the twelve parishes in the island, according to the proportion fixed by the State a few years ago. There is a large out-patient department, and anyone who can obtain a permit from the mayor of his parish has advice and medicine free. The question whether an in-patient can afford to pay the fee of half a guinea is decided by the Board. As there is a girls' home at Grouville, and a boys' home at Gorey, no children other than babies are received. One important structural improvement is to be made shortly. A new lying-in ward, quite separate from the other wards, is in course of construction, and when it is finished the present ward may be utilised for cases of consumption. Before I left the hospital I inquired of the head nurse whether there were trained nurses obtainable on the island. She rejoined that at the hospital they had no knowledge of any, but that inquiries were often made for them.

### The Jersey General Dispensary.

However, pursuing my investigations further, I soon had the pleasure of meeting a nurse whose training is unquestionable. In a poor part of the town there is the General Dispensary, and here I found in charge Miss Piton, who succeeded Mrs. West as matron last year. Miss Piton was trained at King's College Hospital, the National Hospital for the Paralysed and Epileptic, in Queen Square, London. She was afterwards Queen's nurse at Aldershot for four years, and sister at Dumfries Infirmary for eight months. There is an assistant who is not trained. The little infirmary attached to the dispensary contains eight beds, and operations are sometimes performed under the auspices of the resident medical officer, Dr. Spain, who has held the appointment for six years, and attends patients who cannot be accommodated in the infirmary. Four of the latter are non-paying patients, but the other four pay 12s. per week each. The patients are chiefly domestic servants. The number of out-patients is considerable, and the out-patient department is, in fact, like that of an ordinary general hospital.

### District Nurse Given Up.

Miss Piton informed me that there used to be a district nurse attached to the dispensary, but she was given up in consequence of lack of funds. The institution depends entirely upon voluntary contributions, and such support as it receives is chiefly given by English people. The assistant is on night duty, but calls the matron in case of need. Nearly all the accident cases go to the general hospital. Having inspected the infirmary, and observed the unoccupied ground, I asked the matron if the building could be extended, and she replied that if the Committee had the means to build they could enlarge it because they possessed sufficient land for the purpose. With regard to private nurses, she stated that there were two or three on the island.

### Nursing Homes that Failed.

But on this point I succeeded in obtaining an interview with Mrs. Hubert, a lady who for nearly four years conducted a nursing home in Victoria Crescent. Hers was not the first attempt of the kind, she told me, but it was not successful, although her staff of six nurses, one of whom acted as superintendent, were all hospital trained. Some of them, however, were not, I gathered, good nurses, in spite of the possession of good certificates; and that, of course, told against the institution. The previous home obtained a bad name, and Mrs. Hubert herself appears to have suffered from the flightiness of members of her staff. She was, however, satisfied that there is a real need of trained nurses in Jersey. People frequently have to telegraph for them to Southampton or Weymouth, and one of Mrs. Hubert's difficulties, when she had the home, was that she often found her staff insufficient. She did not encourage the idea of private nurses settling in the island on speculation, but she observed that she had often thought it might answer the purpose of one of the great London hospitals to open a branch in St. Helier, providing that the support of the doctors could be secured. Her own experience was that they wanted a lot of looking up.

### The French Sisters.

Miss Piton had mentioned that the Boileau Sisters of Mercy were very kind to the poor, and at Mrs. Hubert's suggestion, I directed my steps to a nursing home in Green Street, carried on by the members of a religious order. This I found to be a delightful abode called The Limes, close to the sea, with a beautiful shady garden. The rev. mother of the "Etablissement S. Marculf," for whom I inquired, and who together with one of the sisters showed me over the well-ordered home, explained the principles on which it is conducted. There are six sisters, all of whom are trained according to the French system. Their work, which has been going on for a couple of years, is of a twofold character. A certain number of persons, not necessarily sick, are received in the home. I saw three, including an English-speaking patient of 86, who seemed in the best possible spirits. The terms are exceedingly moderate, and Protestants are welcomed as warmly as Roman Catholics. The sisters also visit and nurse sick persons outside their establishment. They depend upon the support of the charitable for what they are able to do, and I was not surprised to learn subsequently that these cultivated and kind-hearted ladies are regarded in the town with high esteem and confidence by people who do not share their religion.

### With a Camera in Hospital.

BY A NURSE PHOTOGRAPHER.

There are many hobbies that it is neither desirable nor possible to cultivate when one is undergoing one's three years' training as nurse. Photography, however, is both a

In the Children's Ward.

suitable and eminently possible amusement for a nurse in her off-duty time, and a very great deal of pleasure both to oneself and others can be got out of it. The instinct of the small boy who plants himself in front of the lens at every possible opportunity, is strong in the breasts of most people, and nearly everybody likes being "taken." Besides one can get pleasant remembrances of incidents and faces, friends and patients which are sure to be treasured after the hospital is a thing of the past, and one is perhaps a staff nurse or a sister in some far off spot, or perhaps a private nurse. There is generally some "glory hole" under the stairs, or perchance in the cellars, that Matron, if approached judiciously, will allow to be used as a dark room. Drawing pins and black calico will be found a useful method of excluding any chinks or cracks of white light which may be found to intrude.

The Choice of Plates.

Photography in the wards is not really the difficult matter some people seem to find it. The great point is in the choice of plates. Plates which are used for this kind of work will have to withstand great contrasts of light and shade, and generally a strong cross light. To meet these difficulties, they should be backed, and should be richly-coated plates of the slow variety for preference. I have found Imperial "Sovereign" plates far the easiest to work for this particular purpose. They give extremely "plucky," clean negatives, and have no tendency to fog, which many plates perfectly well-behaved under less trying conditions, will show to a most disastrous extent. Some floors are of polished parquet, others are tesselated Italian mosaic, and the legs of the camera will show a tendency to slide on either. This is easily overcome by fixing a circle of cork on the point of each leg of the tripod stand.

The Best Pictures.

Pictures can be found by the artistic aspirant, especially in the children's ward, and it is wonderful how good the mites are, even down to quite the babies. The portrait of "Our Bogey," aged somewhere about four, and stone deaf and dumb, illustrates this, especially as it was, of course, utterly impossible to make him understand that he must keep still. Except, however, that he would pat sister and then point excitedly at the camera, he was as good as gold, and had there been light enough to give a shutter exposure, a very good picture might have been made of him, with one chubby hand outstretched, a podgy forefinger indicating the strange thing which pointed so mysteriously at him.

A Practical Point.

It is possible, too, that a knowledge of photographic manipulation may come in useful in a more professional manner than if used strictly for pleasure. I mean in the development of X-ray negatives. Many small country hospitals now possess an X-ray outfit frequently used as a valuable aid in the diagnosing of fractures or "foreign body" injuries, and a nurse with a good knowledge of development and suitable printing, may have the opportunity of making herself extremely useful. The usual tendency of X-ray negatives is towards flatness and want of contrast, therefore a plucky negative must be aimed at in development, though great care must be exercised that there may be no blocking up of detail. Some operators use

Men's Medical Ward.

what are known as orthochromatic or isochromatic plates with the object, by means of a calcium tungstate screen, of shortening the exposure. When such plates are used, the developer must guard carefully against light fog, for they are sensitive to yellow, and even red light to a certain

### WITH A CAMERA IN HOSPITAL—*Continued.*

degree. As slow development with a rather weak developer will be found to give the best negatives for this sort of work, the dark room lamp should have a red glass, a canary glass, a green glass, and a screen of yellow fabric. The

Reggie.

dish should have a cover which can be removed at intervals to see how the negative is progressing. If prints are required urgently the negative, after washing for 15 minutes under running water, can be dried by immersion in alcohol, and then placing in a current of air, out of the way, however, of dust or hot sun. A print can then be struck off either with glossy printing-out paper, or on one of the smooth-surfaced papers now on the market for printing by artificial light.

### The Storage of Negatives.

If possible, however, the negative should be thoroughly washed and varnished. It should be kept in a grooved box, and its number and record of details, etc., recorded in a register kept for the purpose. A print of every negative should also be kept in a book, where, if required for future reference, it can be easily turned up and identified. Another good way of storing negatives is to enclose each negative in a waterproof envelope, on the outside of which the details are inscribed. Twenty-five or fifty are then stored together in a box, which should bear a distinctive letter or number; thus if you want negative 22 box D you only have to get out box D and find negative 22 therein. Doctors sometimes require prints for reproduction in medical journals, etc. These should preferably be vigorous in contrast on glossy P.O P.—not too extensively glazed on plate glass—and should be toned to a cold bluish tone. Red reproduces badly. A good plucky print on glossy bromide or some similar paper will also reproduce satisfactorily.

### Matron's Permission.

Films are sometimes used for all photographic work instead of plates. The manipulation is identical, but for drying they should be pinned to a board with drawing-pins,

Our Bogey.

and the drying must not be hastened by immersion in alcohol. It should not be necessary to remind nurses that before any photographs are taken in the wards, the matron's permission should be asked.

### Sketches of Our Hospital:—The Porch.

BY MARGARET E. FOX.

We stand well back from the high road, and hide our face behind a veil of fine old trees, which in summer are riotously green for London. Very likely you do not notice us at all as you ride by on a city bound tram car of a morning or cycle countrywards some Saturday afternoon. That is unless you make a *détour* behind the trees to avoid the traffic, and then you will see we are built of substantial red brick, inlet with windows at equal distances, iron gates guarding our entrance, on the post of one of them being a small blue square thing, on which letters of white give the passer-by some information about street ambulances. There is the ambulance itself, a little farther back, nestling in a corner at the side of the house, its ruddy front clean and freshly painted, and the inscription on it indicative and suggestive of the whole of the work within the walls against which it stands—"Ready for instant use.—Free."

How really ready does not at once appear, for there are times when we are very quiet, though even then full of latent activity. Take a look at us about 3 o'clock on a summer morning. Already on the still drowsy highway beyond the trees, slow, heavily laden carts are rumbling along to the London markets, bearing sweet wholesomeness of fruit and flowers from the hands of the country to the mouth of the City. On the warm, still air their fragrant breath wafts in at the open windows of the nurses' dormitories, and sets them, yet asleep, dreaming of home gardens, with tennis lawns and picnics, and all things which once were as the wine of life, but which now have grown trivial and small amid the daily happenings of our hospital. The solemn half light of the early dawn is penetrating into the wards—those buildings which you can just see from the front near the trees, stretching out on either side of the main building like succouring arms of pity and help, these same arms holding many a poor, crushed, human thing, broken by disease or accident, and comforting it with every tender nurture.

### SKETCHES OF OUR HOSPITAL—*Continued.*

Twinkling jets of light are being extinguished as the dawn creeps on, and the night nurses draw up the blinds to admit the morning. Another ward, nearer the roadway, is beginning to wake up, too. Its windows are large and frosted halfway up, and from inside, float fretful baby wails, as the wee ones awake to consciousness of pain or discomfort. The dark green blinds which have been down all night to shut in the darkness and hush the children to sleep are being raised now, windows are opened, and visions of white-capped nurses are seen flitting up and down the ward. Before long as the dawn gets stronger and the sun brighter, the big iron gates are unlocked, and our day is ready to begin. There are panels of glass in our front door through which the sun makes a perfect rainbow of colours, and through those glass panels you can see into our entrance hall, tiled corridors branching out from it, and a white stone staircase leading up to the nurses' rooms in front of you. To the left is a small room with glass windows, too, for the porch attendant, and to the right a waiting room, furnished sparsely with chairs and registers—a place quiet as yet and untenanted. But even as you look, we are astir. Along one of the corridors, a little group comes, a nurse with a man and a woman, their faces white and worn with a night watch by a sick child, the man, clumsily trying to subdue the fall of his iron-tipped working boots which echo noisily on the floor tiles, the woman weeping silently. The man's face is set, and he stumbles as he walks, his eyes gazing before him with an unseeing look. The woman turns instinctively into the waiting room where he follows her, but the nurse gently urges them both towards the door. "There is nothing more to wait for now," she says, and the man mechanically echoes her words, "No, nurse, there's nothing more to wait for now." The little one they have been watching has just sighed its weary life away, and left them childless. She takes the chain off the front door, and the two go out together into the fresh summer morning. The sad pathetic patience of the poor is never better shown than by these two. He plods on silently to his work and to his labour until the evening, and she returns to their desolate home, both with the fatigue of that anguished night watch behind them, but giving little sign of what they suffer. The nurse sighs, and shutting the door hurries back to her work. Then a maid comes with pail and broom to sweep and wash the already clean floor. Brasses are polished, dust removed, mats shaken. A group of day nurses, fresh looking and bright, come clattering down the wide stone steps on their way to breakfast. A brisk, alert ring, the first for the day, announces the postman with his letters and a weighty bag slung over his shoulders. This, he deftly swings round, and opening, dives into its capacious depths. Some parcels come to light, one or two addressed in quaint spelling to the patients even in these days of twentieth century education. One—a badly packed box of eggs, with albuminous droppings escaping through the soaked cardboard cover—is marked "mary all, ergant" among other items of the address. The postman does not smile, being too used to the humour of his post bag to be always able to perceive it. Depositing his load, he goes briskly on his way, and gives place to the little newspaper boy, who is getting in an hour's work before school hours, and then to the hospital barber, entering familiarly, unannounced, who straightway makes for the men's wards, where he represents the hub of social life to most of the patients.

From this time onwards, the tide of human nature ebbs and flows in and out our porch door without much intermission. People come on all sorts of errands—they want the doctors, the secretary, the matron, the nurses. They clamour for beds, money, bath chairs, work, certificates. Cabs drive up, bringing applicants for admission, or to take patients home, though all are not so fortunate as to be carried on other legs than their own.

Towards midday, a diminutive boy of some twelve years, comes in with the message that he has been sent to take Mr. Hardup home, and he displays a postcard from the ward sister, authorising Mr. Hardup's dismissal. The message goes round to the ward, and the boy waits. Mr. Hardup is notified of the fact that someone has come to fetch him, and laboriously gets ready to leave. He is a huge red-haired young giant of twenty or so, with a recently-mended fracture, and what is technically called a "plaster leg." He moves heavily on his unaccustomed crutches "with measured beat and slow." Thump! thump! The ward sister helps him patiently along the corridor to the waiting room, where he pulls up aghast at the sight of the boy sent to fetch him. "Why aint Jim come?" was his greeting "You aint no good."

"Jim's got a job of work," his brother replies; "I'll help yer, Bill."

The ward sister smiles as she notes the contrast between the two, and bids them wait a moment while she goes to fetch a porter to pilot Mr. Hardup and his brother to a tramcar, and, as good old Pepys has it, "so home." The tide moves on—now ebbing, now flowing, but chiefly in full flow. Early afternoon brings arrivals of a different type, two or three men, keen of eye, alert of movement, cultivated intellect in their every tone, who enter without any preliminary ring and sign their names in a book that lies open on a table just within the porch. They are the physicians and surgeons on the visiting staff, and as if by instinct the resident medical officer appears on the scene at their entrance, followed by the head nurse, and they all go down the corridor together, with a steady look and firm step that means a long afternoon's work before them. Patients' visitors arriving to see certain of their friends on the "danger" list, are now kept waiting what seems to them a wholly unconscionably long time.

"Doctors in the ward. Can't go in yet," is all the reply they get, as they watch the slow moving hands of the hall clock, crawling round its face, and ask the porter from time to time how long they will have to wait. A shy girl of fourteen sits there quite two hours, almost without moving. Her mother is in the hospital, and this afternoon she is undergoing an operation. Her father, himself obliged to go to work, has deputed her to come and ask if the operation is successful. She waits there nervously. How long the doctors are! Nurses come in now and then, dressed in outdoor uniform, and sign their names in a big book on a desk by the side of her chair. She wonders why they do it, and as the book is so very near her, she reads all the names on the long page, and is much mystified. The slow hall clock strikes five—six, and still the doctors do not come along. Then a feeble ring at the front door bell, and her father joins her in the weary wait, tired, dirty, on his way home from work. She is glad to see him, for she is getting nervous, and they whisper together for another ten minutes or so. At last, welcome sound—voices and many steps in the corridor, and a little group of men begin to put on their hats in the porch. "That's him, father," says the girl earnestly, "that's our doctor! I see him."

"Beg pardon, sir, for interrupting," the grimy working man rises suddenly and approaches the group through the open door, "but could you tell me how my wife is?"

One of the surgeons—a tall man with a keen eye—pauses and looks doubtfully at him; the head nurse, who is of the group, says something to him in a low voice, and the surgeon comes forward, his manner reassuring, his smile kind.

SKETCHES OF OUR HOSPITAL—*Continued.*

"Going on all right. Yes. The operation has been performed. Quite successful; and your wife is well—so far. You can call again to-morrow morning."

"Thank you, sir," and with a curious light in his eyes, that somehow seems to irradiate the grimy face and make it beautiful, the man and the girl turn to walk away, but at a whisper from the child he stops again.

"I suppose," he says, somewhat falteringly, "I could not see her, could I, sir?"

"Better not to-night," replies the surgeon, without looking at him; but the nurse, catching a great wistfulness in the expression of the shy girl, and divining her exceeding desire to see her mother, spoke again.

"Could we not make an exception, sir, for once?" she asked softly; "that little girl has been waiting such a long while."

"Just a minute, then." The tone was abrupt, but accompanied by a not unkindly glance; and full of intense gratitude the small girl and her father follow the nurse down the corridor for the promised glimpse. The surgeons depart, the residents go whistling to their room where a cheery forecast of dinner is in the air, and the porter relaxes his rigid attention and sits down to read *Tit-Bits*, keeping, however, a watchful eye on the door meanwhile. By-and-by a trim maid comes to relieve him from duty, and he departs, with a great yawn, for a smoke and some supper. It is a long light evening, and the gas is scarcely needed before it is time to put it out again, and lock up for the night.

Then the last post for the day comes in, and bright-faced nurses, just ready to go off to bed, linger round the door, as the letters are sorted and delivered, some—and these wear mostly the garb of the probationer—with a touch of wistfulness in their gaze as they wait and ask, "Anything for me, sister?"

"Not to-night, nurse! Cheer up. Yours will come in the morning. Good night."

"Good night, sister!" and the little band melts away, the faint disappointment chased into the background by the hope of what the morning may bring.

So the door is locked and the lights go out.

"Good night!"

### Sponging Typhoids in America.

BY A GRADUATE AND SPANISH-AMERICAN WAR NURSE.

HAVING considerable experience in the care of typhoids, and having had typhoid myself while acting as probationer in the Erie County Hospital, Buffalo, I thought that HOSPITAL readers might take an interest in the American methods in which I have had the advantage of theoretical, practical, and, shall I say, physical experience.

### "STANDING ORDER" AND "DAY ORDER."

It is usual for the medical man to leave what we call "standing orders" on every ward—the same idea is carried out in private practice—these orders must be written in books provided for the purpose. The orders for courses of treatment are entered in the standing order book, and also the necessary prescriptions, for the nurses study materia medica, and compound most of the doses. Infusions, decoctions, syrups, emplastrums, and ointments are prepared in the laboratory or drug department, where also the powders are weighed and prepared, though in some small hospitals the nurses weigh powders and fill capsules. The nurses are not expected to carry out verbal orders at any time, so a smaller book is provided for what we term "day orders." This arrangement prevents mistakes, saves a lot of time and unnecessary talk, and if the nurses are very busy the doctors just write their order without interrupting them. Of course the head nurses and senior assistants make rounds with the medical men at least twice a day, when the nurse gives reports and receives instructions at each bedside. The standing orders for a typhoid are:—For temperature 102°, 102½°, or 103°, plunge every two or three hours, temperature of water to commence 85° to 75°, reduce to 65° or 60°; for temperature 101° to 102°, sponge; temperature 100° to 101°, alcohol rub. Precede and follow each plunge by whisky or brandy ℥ss in hot water.

### CLOTHING REQUIRED.

With regard to clothing; so long as plunges and sponges are necessary, it is usual both in hospital and private practice to dispense with everything heating, consequently the patient lies between sheets, with no other covering. A nightgown is prohibited because of the exertion of removing and readjusting it; though if a patient specially requests that he or she may have a gown, a short gown open down the back is permissible. The mattress should be covered with a mackintosh sheet, then put the patient between blankets, and remove personal clothing, if any. It is quite unnecessary to explain how to effect preliminaries as this is meant to interest trained nurses, or nurses in training. Bring to the bedside a foot-tub about half full of water, warm, tepid, or cold, a wash-basin with pieces of ice, and pure alcohol or other spirituous preparation. As few people's skin can stand pure alcohol for any length of time, it should be diluted to about 50 per cent. Several towels and compresses will be needed. Compresses save handkerchiefs and sponges, being cheap, can be burned, and so prevent spread of infection. Have a small basin with ice or very cold water, and a compress with which the patient's face may be frequently sponged, and the compress or an ice-bag should be kept on the forehead while sponging the entire body. It is well to place another compress at the nape of the neck; these prevent to a great degree temporary cephalic hyperæmia.

### MODE OF SPONGING.

Next wring a towel out of the water and place it the full length of the chest and abdomen, wring out others and wind one round each arm and one round each of the lower extremities, and a sixth along the back. Speaking from personal experience, the last is not very comfortable to lie upon. Regulate the temperature of the water by adding pieces of ice. It is usual to reduce the water temperature during the sponge. The towels must be frequently changed in the same order till the surface is quite cold (by the time the back is done it will be found that the towel on the chest and abdomen is quite warm). When the surface is cold—usually between 20 and 30 minutes—the towels will be removed and the patient rubbed all over with spirits. As this is done by hand the alcohol or spirits is most convenient in a pudding-basin. In the Erie Hospital towels for drying purposes are not used, but the patient is left wrapped in blankets for about 20 minutes, and, if necessary, hot-water bottles are put to his feet. Rubbing with spirits has a drying effect, prevents catching cold, and is stimulating. It is desirable to avoid friction, which drying with towels is likely to produce. When the patient is rested, feels comfortable, and his teeth stop chattering, the blankets are re-

### SPONGING TYPHOIDS IN AMERICA—*Continued.*

moved, and he is left between sheets, unless the doctor orders blankets, or the patient's condition indicates the need of them. When the patient's temperature does not call for a sponge he is rubbed all over, for about 20 minutes, with spirits. This is not necessarily done between blankets, it is more for comfort, and to arrest the tendency of the temperature to rise, so an occasional sponge is omitted, which is restful to the patient, and he absorbs cutaneously a fair amount of stimulation. Rubbing with the hand is always gently downwards, rather following the course of the arteries —from, not to, the heart.

### An Ice-rub.

What is termed an ice-rub is given by wrapping a piece of ice in a towel and rubbing all over till the surface is cold. The patient must be between blankets and otherwise treated as in sponging. We tried ice-rubbing, but results did not prove very satisfactory, though patients like it as a novelty. Some nurses appear to think hot-water bottles are not placed to the feet while sponging them with cold water. On the contrary, the soles of the feet or the feet are rarely sponged at all. If they are warm enough to sponge, why apply heat? and if cold enough to apply hot-water bottles, why sponge? In giving packs, most books on nursing advise that the feet be left out. Sponging thoroughly the arms and legs of necessity cools the hands and feet. Indeed, the object of the ice-coil is to cool the blood as it leaves the heart. It also cools the venous blood returning to the heart by the vena cava. By this means each systole and diastole disperses blood slightly cooler than its predecessor for general circulation. So we get a reduction of temperature throughout the entire system without applying any manual treatment to the extremities. When sponging each part is uncovered separately; first the arms, next the chest—cover this with a towel while sponging the abdomen—then each of the lower extremities, and finally the back. Should the patient be troubled with incontinence, pads of common batting covered with butter muslin are made, which can be removed and burned.

### The Question of Covering or Uncovering Patients.

While in charge of typhoid wards in Jacksonville, Savannah, and Huntsville, I learned a diversity of ideas both from nurses and doctors from many of the States. Some of the nurses sponged under the blankets. In such cases I many times noticed the scarlet hue mount to the very brows of young soldiers of all classes in life and society. I think they prefer that each part sponged should be uncovered; then they can see what the nurse is doing and feel much more comfortable, and take quite an interest (provided they are not too prostrate) in the progress of the sponge. I, too, like to uncover each part and see what I am doing. To my idea it is much more dignified, less suggestive, and decidedly less embarrassing, both to nurse and patient. However, I am only a unit.

### Collapse.

It sometimes happens when sponging that the patient shows symptoms of collapse. If the nurse be observant, she will see the finger-nails assume a cyanotic appearance, and watch for other symptoms; or the patient may have a chill. In hospitals there is usually a routine treatment, with which the nurse is acquainted, and a sister nurse can find the doctor. In private practice one may be two or more miles from the doctor and should therefore secure from him previous conditional orders. In any case the nurse should stop sponging. Apply heat externally, with blankets and hot-water bottles, and internally by hot brandy or whisky and water. Coloured people I have noticed seem more susceptible to cold water than white people, and there is an inclination for the temperature to rise again more quickly after treatment.

### The Heaven-born Patient.

He was unfailingly cheerful. His philosophy was of the sort that always sees the turnings in the long lanes, the silver linings in the dark clouds; there was something infectious about his unfailing brightness. The ward seemed a different place after he was admitted to it. He had a cheery word for everyone, and yet he was almost always in pain—sometimes severe pain.

"There's others worse than me," he used to say. "I can grin and bear it. I was always one to grin."

Yes! There was the thing in a nutshell. He was always "one to grin." Never was a patient more considerate for those who were nursing him; he seemed to try only to ask for things at the most convenient times, and if the ward was very heavy, and everyone extra busy, he would lie and wait patiently rather than add to the general rush.

He was not like some patients who seem to choose the very busiest moment to ask for a drink, or who wait until the nurse has just sat down to roll bandages, before calling from the other end of the ward. He was more often than not reproached for not asking for a thing directly he wanted it, and his answer was always the same.

"Why nurse, o' course I wan't goin' to trouble you just when you'd sat down, you've enough to do with your feet as it is. Bless you, I could wait!"

He was a most contented soul, and grateful for every tiny kindness shown to him.

"Nobody don't say nothin' 'gin 'orsepitals when I'm about," he said "they're fair beautiful; and them as looks after yer in 'em is more kind than you could ever imagine."

A grumbling neighbour in the next bed growled out that nurses was paid, and 'orsepitals was just run for the good o' the stoodents and he didn't see nothin' much in them to be so grateful for.

"Don't yer now?" and our Heaven-born patient looked round contemptuously, "Nurses paid, indeed; d'yer think any pay makes up for the work they're doin'? Talk a lot o' rubbish about 'orsepitals bein' for the stoodents, why 'ow much do you and me pay for all as is done for us? Strikes me the least as we can do is to let the stoodents learn off of us. And ain't what the stoodents learns off of us goin' to make em good doctors for everybody? I'm willin' enough as they should learn all they can off o' me. It's the least we can do in return for all as is done for us 'ere."

He never made a fuss over anything, and he was the most philosophical creature imaginable.

"Life ain't long enough to be always a grizzlin' over it," he said; "take things as they come that's my motto, whether they hurt or whether they don't—take 'em as they come and make the best of 'em."

When he was too tired to do anything, he lay watching the busy life of the ward, beaming upon us all. It was a pleasure to pass his bed and see that cheery smile. When in the long night watches he could not sleep, he never grumbled. "I've been a jolly healthy sort o' feller all my life," he said, "its only fair as I should have my bit of a turn at bein' took bad. 'Tis all in the day's work ain't it, nuss—things ain't all goin' to be beer and skittles like; there's always a summat—and if you take it right way up 'tis a good summat for yer in the end!"

He was indeed a Heaven-born patient.

### Words of Advice to Nurses.

BY ESTHER H. YOUNG.

### LECTURE I.—HOSPITAL RULES.

There are some questions of manifest and wide importance which do not necessarily come within the scope of lectures delivered to you in your various hospitals, neither are they included in the rules belonging to these institutions, but they are, nevertheless, questions concerning matters about which you may be glad of advice—possibly of teaching—and I trust these few words may be a help to you. If they are, will you in your turn try to help others, and the nurse who in these words speaks to you, as well by remembering her in your prayers?

Nurses enter on hospital life with such very different motives. A young woman has a step-father or step-mother, so determines to leave home and go in for nursing; another comes to grief over a love affair, so tries hospital life; another has had some experience of nursing at home, so is "a born nurse," or she is obliged to earn her own living, and chooses nursing as a profession, or she has failed in something else, and now wishes to "try nursing!"

There is no harm in any of these reasons, yet there is a higher motive to be added to these (or possibly to be the one and only reason!) and if you do not start with this, try to gain it. The right motive with which every woman should attempt to enter the nursing profession is a real desire to tend and care for sick people—God's sick folk, His creation, made in His image and likeness. The work must be done in His name, for His sake, and with His help all through; therefore to the very best of your ability. You must have a very real love for your fellow-creatures, and an absolute forgetfulness of yourself in the earnest wish to help others. With this motive a woman will make up her mind to give up a great many things, in themselves not wrong, but unsuitable for the life of a nurse, because a nurse must devote herself to her calling, and be ready, if necessary, to give up things that are quite compatible with the life, but which, for the time being, are out of her reach. She may even have to give up, for a time, valued religious privileges. Let her remember that in every way her life should be one of real self-sacrifice She must consider the matter thoroughly before attempting the work, and then she will devote herself to her calling, and endeavour to make it her life work.

The hospital nurse should try to keep in touch, as far as possible, with the outer world—with the events of the day. As I hope to point out, when we consider the subject of carefulness, a nurse should try to keep up, if possible, her art, music, needlework, reading, etc., and (in reason) bicycling, tennis, or any other amusement; but during her training, and probably afterwards, too, these must be rather secondary considerations.

Nurses' work should never be made to give way to their own amusements, but in their "off-duty" time they should be encouraged to go to their own homes or to visit friends. Organising recreations for themselves is certainly to be discouraged, and so also any plans for amusement that may tend to bring about any undue familiarity with the medical students with whom they work.

Now let us consider, first, the new probationer. She enters hospital, it may be a large one, or small—but it is all "so different" from anything she had expected! She is nobody—everybody's junior—and it is taken for granted that she knows nothing. She has been living a "home life," or—as she has probably said during her interview with the matron—"doing nothing!" Here she has to get up at a fixed hour, sweep, dust, polish, make beds, etc., etc., and she must be ready and willing to learn, for all these things have to be done well.

She says: "This is not nursing." Yet it is the groundwork of nursing, and must be learnt very thoroughly. Further, all these things must be done in the same spirit in which the more interesting part of the nursing is to be done later. Therefore, when you sweep, leave no little heaps anywhere; when you dust, don't flick the dust about, but take it off, and shake your duster out; when you scrub mackintoshes, be sure you do not leave a speck of dirt; when you polish, make "brights" really bright! All these little things tend to the well-being of the patients, and are really most necessary parts of the work of nursing. Too often the new "pro." looks upon all this as waste of time—work to be "got through"—but a good nurse must first be a good housemaid.

Give your mind to each little thing, and do all without a murmur. If you find that you are required to do a certain thing, even when you know it to be the duty of another probationer, do it without making any difficulty. Never argue with your superior officer, but "do the next thing."

Remember, too, Who it is who "hath placed it before you with earnest command."

Whatever your work, or profession, you should always let your religion be a part of your life, and not something kept for church and Sundays. And surely nursing and goodness should go hand in hand.

Maintain a high ideal as to what a nurse's inner life should be, and from the beginning keep this ideal before you; aim at it, and however irksome your daily duties may seem to be, do all things thoroughly.

All this applies to the groundwork of your training. Let us now consider some hospital rules, written and unwritten, and hospital etiquette generally.

First, I should like to point out that when a nurse is found fault with, her frequent excuse is: "Oh, I didn't think!" or "I didn't mean to break a rule," or "I didn't intend to do anything wrong." "You didn't think!" as though the fact of not having thought was quite sufficient excuse!

One Sunday afternoon, some years ago, I heard an address given to some school children on the subject of thinking. They were told to think, not only while they were praying, and after they had risen from their knees, but also before they said their prayers. They were to prepare themselves by thinking beforehand, for a moment, in Whose presence they were, and to Whom they were about to speak.

Surely we, too, may well apply this habit of thinking, not only to our prayers, but to our work, and, in fact, to everything connected with our daily life.

I remember, also, that this teacher said to the children: "At many tea-tables scandalous tales could be stopped if only people would think of the mischief they were doing." Surely, in passing, I may remark that in hospital there are many scandalous tales that need never be told if the would-be teller would only "stop to think!"

To apply this to the rules. If you would think more, you would offend so much less in this particular way. More than half the times when you disobey, the wrong-doing is caused by want of thought; so, if you are in doubt as to whether it is right or wrong to do so-and-so—think! This will probably lead you on to ask about the matter, and perhaps much trouble will be avoided. It is just the same, you know, in your work in the ward; so many of these intensely stupid and senseless things which probationers, and even head nurses, sometimes do arise solely from want of thought.

### WORDS OF ADVICE TO NURSES—*Continued.*

Thinking is so necessary in a nurse's whole career that it will be good for you to practise the art in relation to the necessity for keeping Hospital Rules. Do I hear someone saying: "I never think. I can't—there's no time"! My dear nurses, you have often been told, "There is no such word as 'Can't!'" Please say instead: "I'll try!" Try to practise the habit of thoughtfulness about little things, just as you would practise your scales if you wished to be a good musician. Here again we must aim at perfection!

In most hospitals there is a rule to the effect that "probationers must obey the matron, and staff nurse, or sister in whose ward she is working," etc. Obedience is one of the first rules a probationer must learn, for unless she makes up her mind from the moment she enters hospital, to be absolutely obedient to those in any authority over her, she will never be fit to be left in charge of a sick person. Neither would she be fit to live in any institution unless prepared to yield implicit and steady obedience where it is due.

Also, she must be quiet and orderly in the wards and all over the hospital, even, too, in her own room, cultivating the habit of quietness so essential in a good nurse. Creaky shoes must, of course, be avoided, as also high heels or wooden ones, jingling chatelaines, or chains of any sort.

This quietness is not only essential to nurses for the sake of their sick folk, it is also necessary for the well-being of any set of people living together in a public institution—out of respect for each other, and themselves, too.

Again, all nurses must be punctual, at all times, all their lives—so the new probationer must learn or cultivate (as the case may be) this very necessary habit, from the time of her entering hospital.

Be punctual going on, and be punctual going off duty; be punctual at meals, lectures, classes, etc. I need hardly say that all medicines, food, stimulants, etc., must be given at the time ordered, and with exactness and care—this is learnt in each ward—but it will be well for you at the outset to remember that in hospital life you must be punctual always, and the cultivation of this habit on every occasion will be a great help to you in your training.

If you remain in your ward after your time, you are just as disobedient as if you went on duty half-an-hour late. Hospital hours are arranged for you by those in authority over you, and you should clearly understand this point, *i e*, that you disobey your matron if you ought to leave the ward at 2 o'clock, but remain on, without leave, until 2.15, or later. Though she knows nothing about it, you are disobedient. This rule is perhaps more often broken at meal times, when there is a rush, but unless you are really detained by the sister of the ward, you have no right to remain at your work.

The subject of uniform is a wide and rather difficult one to deal with, as hospitals vary so much in their arrangements about it, and matrons have such diverse opinions; but whatever the rule may be, obey it!

Avoid decorations, whether indoors or out; fancy waistbands and buckles are out of place; jewellery is very often forbidden (it is, of course, unsuitable, and should never be worn with the uniform of a nurse); flowers, too, are most objectionable, and even when they are not forbidden, it is generally known that those in authority object to this adornment. I do not think it is a cleanly habit (flowers are apt to drop about and look untidy), and it is cruel to the flowers. Why not be kind to them, and put them in water?

Now let us think about your life in your house (if you have a separate building), or in your cubicles and sitting-rooms.

Here, too, you must aim at perfection! Neatness and tidiness are absolutely necessary for the welfare of your patients, and you should practise these duties in your own rooms, and keep them neat and tidy. Punctuality is essential; practise it the first thing in the morning, and make up your mind that however early the breakfast may be you will never once be late. Aim at this point, and when your time of training is over, see how far you have kept your rule.

However early the breakfast may be, and though you may have to attend prayers, never—yes, never—go to breakfast without having said your own private morning prayers. Don't make the public ones "do" instead, or gabble your own as you run downstairs arranging your cuffs!

At night, probably there is a fixed hour for "lights out." Perhaps you think this does not matter, and most likely, if your rules say that after the lights are put out silence is to be observed, you think this, too, is "rather hard," but the rule is made for your own good, and it is your duty, for your own sake, and for the sake of those around you, to keep it very strictly. Indeed, if you sleep in cubicles, it would be better if you would keep silence for a short time before "lights out," to enable those who wish to do so to read and pray in quiet. I am sure you naturally talkative people do not intend to be unkind or selfish. You do not wish to come between a soul and its God, yet by your thoughtless chatter you do hinder others in this way.

And then, after the lights are out, don't light up stray bits of candle. It is an act of disobedience, and, moreover, a very dangerous one, for you may go to sleep and leave the candle burning, thereby endangering the lives of many. There have been some very serious accidents caused by this heedlessness; and I am sure a warning is necessary.

Probably there is a rule as to keeping valuables locked up. In any case, it is your duty to be careful, and it is advisable not to keep much money or any valuable property in your cubicles or bedrooms.

There is usually a rule that "stimulants may not be kept in your rooms, and are only to be taken by doctor's orders, or at meal times only." If this is not a rule, make it your own plan. I have no hesitation in saying that nurses are far wiser if they abstain altogether and always from stimulants. Their temptations to exceed are great, both during training and afterwards—especially in private nursing—so that I am sure the "better way" for a nurse is to abstain altogether.

Dormitory rules, or home rules, are generally few in number, and very simple, but they are absolutely necessary for your well-being, and for the hospital generally; and you will do well to be very careful about keeping them. My advice to you is that you should read them over occasionally, and think about them, and I am sure you will find that they are not so hard to keep as you may imagine.

In concluding this lecture, I would urge you to persevere in your efforts to keep your rules. Do all your rough, uninteresting work in the best way. Do not get tired of it, but "stick to it!" Don't give in, and don't say: "It's no use trying!" Determine to do your best.

I add this little story by way of illustration. It is very instructive:—

There were two little frogs. There were two bowls of milk. One fell into one bowl, and one into the other. One worked his little feet up and down for a little while, to keep himself alive, but he got tired, said: "It's hopeless, no use trying!" and gave it up. In the morning he was a dead little frog, lying at the bottom of a bowl of milk. The other little frog swam round and round, on and on, got very tired but "stuck to it," and never gave in. In the morning he was a live little frog, sitting comfortably on a pat of butter!

### Beyond the Seas: The Lepers' Home at Jamaica.

By the Matron of the Kingston Lunatic Asylum.

My friend, the Matron of the Kingston General Hospital, and I arranged to take a day's outing together. The place we selected in which "to spend a happy day" was not likely to attract the madding crowd. Yet for us the Lepers' Home had great attractions, which were more than realised.

It is a government institution where the lepers are now detained who would otherwise be wandering vagrants, subsisting on whatever charity compassion for their terrible affliction might wring from the public. That this segregation of the unclean from the community is a necessary and wise system nobody can deny. There are differences of opinion as to the extent of the contagious or infectious nature of the disease, and "who shall decide when doctors disagree?" But no one denies its hereditary character, especially from the maternal side. The medical officer was our cicerone. We drove with the doctor to the Home, which is not far from Spanish Town. We soon saw that he is much liked by his patients, who responded to his cheery inquiries with requests for small indulgences and changes of diet, which were granted whenever it was possible.

### The Wards and the Church.

The wards are one-storied, detached, painted wooden structures, standing in a well-kept irrigated garden. They have polished floors, are clean and well-ventilated, and the red blankets on the beds have a cheerful effect in the rooms. There was no offensive odour anywhere. A shelf over each bed holds those personal belongings so treasured by patients. The medical officer would like boxes under the beds instead, but I think shelves are preferable, though lockers are tidier. The natives are so fond of hoarding up rubbish and stale food, which attracts ants, etc., and shut-up receptacles are not so easily supervised as open shelves. We were shown the pretty well-fitted church, which has a coloured-glass window over the altar and a tiny vestry all complete. This little edifice was the gift of a lady visitor. Previously there had been no special place of worship, divine service having been held in the dining-hall. The kitchen is rather small and inconvenient. Dinner, consisting of meat and vegetables, was being distributed when we looked in. The medical officer is trying to get boilers added in the laundry so that the clothes may be sterilised—a wise precaution I should think. I must explain to English readers that clothes are not usually boiled here. After being washed they are spread on the grass and the tropical sun does the rest.

### Lepers on the Stage.

We saw the schools, where the better educated inmates teach the others. The partition is removed when they have entertainments. These are got up usually by the patients themselves. The stage-manager was once a schoolmaster. He showed us with great pride the theatrical wardrobe. The last play performed was an adaptation of Shakespeare's "Henry IV."! I do not know which struck me more, the ludicrousness or the pathos of it. Fancy these poor black or coloured creatures, disfigured by a loathsome disease, their features altered, their limbs maimed, with sores and growths on faces, hands, and feet, talking Shakespeare by the yard (as the medical officer assured us they did), decked in mimic crowns and robes! One very funny thing, though easy to understand, is that they think no play complete without a doctor. The one they all look up to, who has the most influence on their lives, is the medical officer, so they suppose that a doctor is absolutely necessary. Now the profession was totally ignored in "Henry IV.," and they consulted with the medical officer about what was to be done. He suggested that the schoolmaster should write in a doctor's part. So an extra character named Dr. Martin was represented in their play. Their fêtes sometimes last three days. They present a list of what they want to the medical officer, and, as fast as possible and funds permit, the programme is carried out. Sometimes they have cricket matches—red bows against blue. They have also got a billiard-table.

### The Farm.

We saw the farm, where the patients are allowed to grow vegetables and fruit. They take great pride in their kitchen garden, and showed us sweet potatoes, bananas, etc., of their own growing. In fact, recreations and occupations of all kinds are encouraged. Not only must this be of incalculable benefit both physically and mentally to the patients, but the medical officer has been enabled to reduce the expenditure to the extent of about £1,000 per annum. This is no small matter in these times of financial depression. The entire staff for 178 inmates, mostly ill or crippled, consists of the medical officer, who also has charge of the General Hospital, the police force, etc., the superintendent (who is also the dispenser and clerk), the matron, five nurses and three labourers.

### Improvements Needed.

So far I have scarcely written anything but praise; but there is room for improvement in two things. The first is a minor point. I cannot help thinking that the nurses should wear uniform. The cost is very little when the nurses make their own and the washing is done on the premises. The contrast in the appearance of the Kingston nurses—whether at the asylum or the hospital—with those at Spanish Town is marked. We find, too, that the patients look up to them more when they wear a distinctive dress, and the nurses have more self-respect when they are always in clean and tidy dresses and aprons. But it is the bathing system which is most in need of alteration. With us every patient who is in good health has at least once a day a bath under a shower, and the water runs off down cement-gutters to the drains. Consequently each one has clean water. The sick ones have warm baths. But in the Lepers' Home they have large plunge baths in which many bathe in the same water. This is not the fault of the management, but because there is not sufficient pressure to give each one a clean bath.

### The Disease.

I should like to say a few words about the disease, as it is so little known in England. The medical officer thinks it is not the leprosy of the Old Testament, which is described as being "white as snow." But it is identical with that now existing in the East and West Indies, Palestine, Norway, Iceland, Greece, Madagascar, Madeira, etc. It does not seem to be so contagious as it was once considered, though, like all diseases caused by a bacillus, it is so to a certain extent. The strongest proof possible is this:—Some years ago sufferers from yaws, another loathsome tropical disease, but curable, were detained along with the lepers. At that time many thought that the diseases were akin. Afterwards the cases of yaws were removed, and not one has returned as a leper, though some had lived for years with them. For years we had in the lunatic asylum two cases of leprosy—one of each sex. Neither patient nor nurse caught the disease. Of course they ate and slept apart from the rest, and their

### THE LEPERS' HOME AT JAMAICA—*Continued.*

linen, utensils, etc., were kept separate and disinfected, but they mixed with the others in the airing courts. They died about two years ago.

### Father Damien.

Cleanliness is said to be next to godliness, but they seem far apart sometimes in the cases of devotees. Speaking of Father Damien at Molokai, the medical officer said that he was an ascetic, and in his case asceticism spelt dirt. Hygiene and sanitation were not studied in his cult. He would not adopt the simplest precautionary measures. When visiting the patients at meal times, for instance, he would bless their food, and partake of some from the same bowl into which they had dipped their fingers! In his exalted state of mind he doubtless felt that he was a willing martyr who had sacrificed himself, and would probably have felt disappointed if the offering of his life had not been accepted.

### Cures Rare.

Leprosy, though sometimes slow, is fatal. So-called cures are so rare that they are usually regarded as not having been *bonâ-fide* cases. The tubercular form lasts on an average eight or ten years—the anæsthetic kind may linger for twenty. In the earlier stage of the latter variety blebs are formed on the skin, which burst, leaving behind ulcerated and inflamed surfaces, and these, when healed, leave white, smooth, depressed scars devoid of feeling. The hair drops out, and fingers and toes are often lost through death of the parts. Tubercles, which occur chiefly on the face, hands, and feet, vary in size from small shot to a nut. There is great alteration in the features, the expression becoming leonine through the frowning eyebrows and the ruggedness of the face. The anæsthetic form sometimes exists alone, but the tubercular variety is always combined with the former.

### Yaws.

As I spoke of yaws I will mention a circumstance told me by a doctor. A woman sentenced to three months' imprisonment was found to be suffering from this disease. What was to be done with her was the problem. She could not be admitted into prison in this condition, neither could she be set free. So she had to be kept in a hut, and provided with a warder-servant. By the time her sentence had expired the disease was cured. Thus having to pay the penalty of her crime was a piece of sheer good luck for her, as she was boarded, lodged, and nursed gratis, when she would otherwise have had to suffer great hardship. What usually becomes of these cases I do not know. There should be some special hospital, for I believe that the disease is common, especially in the country parts, but there is not.

### An English Nordrach.

BY A PATIENT NURSE.

An "Open-air" Sanatorium. How often had I heard and read of such places with a nurse's interest; but that I should be condemned to one for three months was a thought that had never entered my mind; yet such was the doctor's verdict which I heard with a sinking heart. If a bad thing is to be done, do it promptly, has always been a motto in which I have believed, and I was thankful that in this case I was enabled to act upon it, and that a week from the day when the warrant for my imprisonment was signed I was on my way to one of our English "Nordrachs."

Refreshed by a long drive in an open carriage after a tiring railway journey I arrived at my destination quite energetic enough to present a bold front and to persuade myself into thinking that the new life was not so much to be dreaded after all. A group of patients, presumably gathered for afternoon tea in the garden—but, as I learned later, in reality to see what the new patient was like—took a good look at me as I went into the house.

### The Question of Meals.

A kindly matron led the way to my little bedroom with its wide open window and wicker couch, where I was glad to rest until supper time, and this introduces me at once to the all-important subject of meals. For abundant feeding I was of course prepared, and I dreaded it. Abundant fresh air and draughts no one who has lived in hospital would dread, but deliberately and conscientiously to eat far more than you want and more than you have hitherto thought compatible with good health and good manners is most disgusting! On the whole it is not so bad as I expected. One good thing is that three meals per day at 8 A.M., 1.30 P.M., and 7.15 P.M., are all that is required of us; there are no wearying glasses of milk nor cups of beef tea which daunt one so at home. A pint of milk at each meal is our necessity rather than our allowance, and good it is for that patient who has never said "I can't take milk, it does not suit me." Good, too, is it to have been trained in hospital to eat all sorts of food which at home one would turn from with disgust, for in a sanatorium we must eat what is given to us be it suet or sago or any other of our pet abominations What is more, we must eat all that is given to us. "Are the patients never sick with such a quantity of food?" I asked our little chambermaid who brought my breakfast to my bedroom the first morning after my arrival. "Oh yes," she cheerfully replied, "but it isn't any use, for they only have to begin over again." Of course we grumble at what we have to eat. We quite forget that our mothers once taught us that it was bad manners to talk about one's food; we are quite as bad as hospital nurses for that, but then there is more excuse for us for our world is very small and our interests are limited.

### Sanatorium Paces.

I said I was prepared for abundant food and abundant fresh air, but there is another law in the life of a sanatorium for which I was unprepared. I was first introduced to this law as on my second morning I ran downstairs in front of the doctor, upon which I was given my first lesson in sanatorium paces. We may never run and we may never walk faster than we should in a funeral procession! Now, I would ask any nurse who has learnt to love the rate at which she went in hospital if this is not a difficult lesson to learn? I am off almost before I know it, but I am not left long in ignorance of the fact that I am breaking rules, for the alert eye of the doctor sees everything, and woe to that patient who does not do as she is taught. She asks that allowances may be made for her—she had so dreaded coming to a sanatorium, and having discovered what a jolly life it is depression had vanished and animal spirits would have their way—but this excuse is only met by the cheering statement—"animal spirits lead people to the devil."

### A Fallow Life.

It is not only that we may not be muscularly energetic but we may not be mentally so either. Before I left home I thought, "I shall at any rate have plenty of time for good

### AN ENGLISH NORDRACH—*Continued.*

solid reading and study," but, alas! solid books are looked upon with disapprobation, and even much letter-writing is discouraged. We must learn simply to lie fallow. It is surprising how short a time it takes to grow accustomed to, and even to thoroughly enjoy, this fallow life. We gasp as we think of the rush of life we have left behind, and are as much astonished that we could bear it as that we could ever bear to sit in a room that has not all its windows "thrown wide open to the day." Wherein exactly lies the pleasure of this inactive life? I ask myself, and I answer, firstly, its irresponsibility. Just to do as we are told; no need to decide for ourselves, our duty being to live to get well, and not to worry about the dark side of life which may have rested too heavily upon us in the past. But the pleasure is not only negative. There is the pure, strong, hill and moorland air so refreshing after the town, there is the beauty of the country of which we learn more and more as our daily walks are lengthened, as our physical health improves, and there is the perpetual interest which the forty odd patients never fail to provide. It is impossible to be thrown as we are into companionship with so many without making new and delightful acquaintanceships and friendships and without finding endless sources of interest and amusement. Neither need this life be a selfish one. It has its happy side, but there is the sad one, too, and much that calls for "a heart at leisure from itself to soothe and sympathise."

### No Trained Nursing.

One thing here has surprised me very much. Of course, if a patient is very ill, he or she has a special nurse; but for the ordinary patient who has fever, and is therefore kept to bed for weeks, there is no trained nursing. As a nurse this gives me reason to question and most emphatically to condemn, but—I am only a patient.

### The Daily Round.

With what regularity our days are lived. Called at 7.15, we dress and await our first visit from the doctor, who tells us how our mornings are to be spent, whether resting in the grounds or taking one of the regular walks from which, be it ever so short, we must not return many minutes before 12 o'clock, which is "rest hour." Before dinner we are visited again, and our afternoons planned for us. Usually we are not sent for long walks in the afternoons, and we are pretty sure to be back for afternoon tea at 4 30, for not only do we love our cups of tea (we have nothing to eat with them), but 4.30 is also the hour for the second delivery of letters—a never-failing attraction. Six to seven is rest hour gain, and before 7.15 we receive our last medical visit. Of course we take and chart our own temperatures four times a day. After supper we stroll about the grounds, gossiping or enjoying the music which comes to us through the open windows of the doctor's room, until at about 9.30 we retire for the night.

### Eccentric Escapades.

Each day is passed just the same, varied only by the weather or by some eccentric escapade, such as sleeping all night out of doors on the lawn, with no protection from the pouring rain, or taking our walks barefoot, or letting our hair down our backs to dry after washing in the fresh breeze—all of which eccentricities and many more besides are heartily encouraged in this sanatorium.

We hardly ever wear hats; indeed on the Sundays when church service is conducted in the grounds, it is quite an entertainment to see the ladies wearing hats—we look at one another and feel we require reintroductions. If we get thoroughly wet we are not encouraged to change our clothes, though I think many of us do, not having sufficient faith to be sure that, though we shall not be more likely to die of phthisis after exposure to all sorts of bad weather, we may not live and groan with rheumatism!

### The Event of the Week.

There is another thing which struck me as soon as I got here—the patients look so well. There are not many who are extraordinarily fat—this was a great relief to my mind—but nearly everybody has a rich red-brown face, which is hard to reconcile with pre-conceived notions of phthisical patients. This fact speaks for itself, especially when one has also noticed the new patients who arrive thin, wasted, anæmic, gradually putting on Nordrach complexions; and one has heard them exclaim on weighing days, "I have gained a pound per day" or "I have gained three pounds in the week." Weighing day affords much interest and excitement to the community. At 8 A.M. there is a tramp of many feet along the corridor, a small crowd collects round the weighing machine, and as the record of each patient's weight is proclaimed there are congratulations or condolences offered from the spectators, and if anyone beats the record there is even an attempt at applause.

How quickly the weeks pass here! I can scarcely believe that six have slipped away since I came. I wonder now why I thought it such a trial to come, and smile as I receive letters of condolence from sympathising friends. "T Bs." are wicked little things, but even they bring good along with them, and I can cordially thank them (let them doubt my sincerity if they like) for giving me a very good holiday and the very interesting experience of life at an "open-air" sanatorium.

---

### A Visit to the Hospitals at Beyrout.

BY A CORRESPONDENT.

There are 120,000 inhabitants at Beyrout, and there are five hospitals; but as it is a large seaport town situated at the foot of Mount Lebanon, the number is not too large. Though they are all meant for the nursing of the sick, yet they are so totally different from each other, and their objects are so varied that it is most interesting to visit them. There is the military hospital, which is the largest; the Prussian, which is the most beautifully situated; the French, which is built on the most modern lines; the Greek Orthodox which has the prettiest and most attractive interior, and the Municipality Hospital, which is the dirtiest hospital I have ever seen.

### The Turkish Military Hospital is an immense building with large grounds and gardens, wide passages, and contains 250 beds. The chief medical officer, Khairi Bey, and the chief surgeon, Munir Bey, speak French perfectly and are well up in all modern ideas. The hospital is very clean, tidy, and well managed. I had sent word to say I was coming to visit the hospital and they appointed Sunday morning for me at 9 A.M. As I drove up to the gates one of the doctors came up to the carriage, helped me out, gave me his arm and introduced me to all the other doctors (eight in number) who were waiting to receive me with a large number of soldiers. This was a great honour for Turks to

### A VISIT TO THE HOSPITALS AT BEYROUT—*Continued.*

pay to a woman, and when I looked back at the days of my probation, when with very rough red hands I had to run forward and open the door for the doctors, the reception I got slightly took my breath away and made me feel somewhat of an impostor. Several soldiers walked in front, and every now and then turned round, bowed low, and waved their hands in the direction they meant me to go, while the doctors walked behind to show their respect for me. I was exceedingly surprised at the order, discipline and cleanliness of the place. The operating room was spotless and contained sterilisers both for instruments, dressings, and the doctors' white operating gowns. When we had done the round I was taken across a long terrace into the reception-room which contained a picture of H M. the Sultan, under which there was a canopy. In the centre and on either side there were armchairs. I was offered the middle seat under the canopy, and on either side, in rows, sat all the doctors, the chemist and the steward, according to their rank, so that by the time they got to the chemist and the steward the seats were by the door. I told them that in English military hospitals we had ladies to nurse and direct the orderlies. They said it was an excellent plan, as no household could be managed without a woman, but that it would be an impossibility for them to have women in a military hospital. Finally, they brought the coffee and I was able to leave, the doctors and officers all following with some twenty soldiers; at the gate the soldiers saluted, and several stood at the carriage door and loaded me with ready-made bouquets of flowers. I drove off feeling quite as if I had been Royalty.

### The Municipality and Prussian Hospitals.

I went straight to the Municipality Hospital, where I was not expected. I do not think I ever saw anything so dirty, so sad, and so unlike a hospital. It is kept by the Municipality funds and is consequently in the hands of the Government and government doctors. Few people care to go to this hospital, except those rejected by other hospitals or discharged as incurable. All sick State prisoners are also sent there.

The Prussian hospital belongs to the Order of the Knights of St. John of Germany. It is nursed by the Kaiserswerth Sisters and is attached to the American School of Medicine. It is the oldest hospital in Beyrout, and is situated on a hill with fine grounds and a lovely view of the Mediterranean Sea and Mount Lebanon. The sisters wear dark-blue spotted dresses with blue aprons and no bibs, white spotted net caps with a white frill framing the face, tied with strings under the chin of the same material. The hospital is understaffed and the sisters work very hard indeed even during the intensely hot summer months. Latterly, a new theatre and a beautiful surgical ward have been built by Dr. George Post, a well-known botanist, and the first surgeon in the country. What took my fancy most in this ward was a dressing-stand on wheels, on which were fixed three glass barrels with tubes for irrigating the wounds, a basin for washing the hands, and a dish for the instruments. In the operating-room they have glass cases for the instruments, sterilisers, and all the modern appliances, and before entering the students are obliged to change their boots for india-rubber snow boots.

### The French Hospital.

For the credit of the French I am glad to say that the French hospital is in accordance with new ideas of sanitation. It is connected with the French School of Medicine supported by the French Government. The doctors are all French and there is an agreement with the Turkish Government that French professors come out yearly from France to examine the students, so that they are not obliged to go to Constantinople to get their degree as is the case with the American Syrian Protestant College School of Medicine.

The nursing is done by the sisters of charity of the Order of St. Vincent de Paul. They are all sweet, devoted women, and give a tone of refinement and gentleness to the whole place. They have very large, fine wards, each separated from the other by a garden, so that each is detached and self-contained, and the air is able to circulate all around. Each ward is dedicated to, and contains a life-size statue of, a saint surrounded by lights and flowers. There is a sister in every ward, and under her she has native women from the orphanage of the same Order who speak French and do all the rough work and superintend the scrubbers. On the male side men work under the sisters. There is a most beautiful chapel always decorated with flowers, while plants and palms meet one in every direction. The maternity block is exquisitely kept, with every possible appliance down to an incubator. But the sisters never attend the confinements, nor even the operations. The operating theatre, from an architectural point of view, is the best in Beyrout. A sister who is in charge always sits in the anteroom, where she keeps all the requisites, and in case of anything being required the surgeon calls out for it, and in the twinkling of an eye she runs in with it. Otherwise, the students do all the attendance with the help of male servants, the latter doing the cleaning up. I never saw anything so well kept as the theatre sister's room, cupboards and drawers. When strictly in uniform the sisters wear dark blue aprons, yet in hospital they may often be seen in white linen ones. Alluding to linen reminds me of the linen room; here, again, I never saw anything more beautiful. There stood the good sisters scenting the linen with jasmine and lavender, darning and mending most wonderfully even the oldest garments. From an English point of view these sisters are not properly trained nurses, but the nursing and work done with such loving hands must be valuable.

### The Greek Orthodox Hospital.

Perhaps the Greek or native hospital is better known as "L'Hôpital Orthodox de St. Georges." It is supported by Syrians of the Greek Orthodox Church. The managers admit patients of all religions and denominations, and are as unfanatical as they can be. There are six doctors, five of whom are natives, and the sixth a Greek. The lady superintendent is Miss Wortabet, who was trained at the Middlesex Hospital and at the London Temperance, and has had a varied experience of workhouse, district, and private nursing. She speaks French and Arabic fluently, and has thus been able to take charge of and reorganise the hospital. The nursing is done on entirely English lines, by native nurses dressed in English uniforms. There is one native nun who is very useful on some occasions and very trying on others, especially on feast and fast days, otherwise she nurses the most loathsome and trying patients with the greatest devotion. The hospital as a building is very fine and unusual in architecture, and at the same time is most hygienic. It is built on the site of the ancient necropolis of Beyrout. The large white marble fountain in the centre of the garden and most of the marble pillars came from the ruins that were dug up from the foundations. There is a large garden in the centre, around this there runs a wide colonnade with marble pillars. This square colonnade connects all the wards, but most of them are detached and are built on the block system. As an interior St. George's Hospital is unquestionably the most

### A VISIT TO THE HOSPITALS AT BEYROUT—*Continued.*

attractive. The operating theatre also contains the best articles and the most costly. The floor is of white marble, the walls are painted white, the operating table is white enamel with handsome mountings and crutches of best French nickel, also the glass cases and the instruments themselves are beautiful. There is a large steriliser for the instruments and dressings—combined—on the new system of dry sterilisation. This has two advantages—one of preserving the instruments and the other of punctuality and time. Everything can be sterilised beforehand, and the instant the surgeon is ready the case is opened and the lid removed to reveal the glittering instruments.

### Treatment of Lunatics.

The lunatic asylum for Beyrout and Lebanon is one of the greatest boons, as the treatment of the insane in this country is very sad, and in fact brutal. This establishment is partly self-supporting, but is mostly kept up by funds from America, England, Germany, and Switzerland. The administration, medical attendance, and nursing is done by Mr. Waldmeier, a German doctor, and German and Swiss male and female nurses. The language is proving a great difficulty, but not an insurmountable one. Some of the girls from the German Orphanage are training as nurses, while the doctor and the others are studying Arabic.

Beyrout being such an educational place, with two schools of medicine and so many schools and orphanages, places it on an entirely different footing from other towns in Palestine and Syria.. Eye and fever cases form a large part of the percentage, but excellent surgical work is done. It is also quite astonishing how very much more quickly the wounds heal here, and reminds one of experiences of the Transvaal war. A peasant on Mount Lebanon was stabbed in the abdomen so badly that all the entrails fell out; he caught them up, put them back, and walked several miles to the nearest doctor, who sutured him up and sent him home, and the man recovered! This is a true story, and I know both the doctor and the man.

### Presentations.

South Africa Refugee Camp.—On the 22nd of August a dance was given in the Refugee Camp, Krugersdorp, Transvaal, to bid farewell to Sister Sutcliffe, who has been transferred to the Refugee Camp, Potchefstroom, Western Transvaal. The staff presented Miss Sutcliffe with a handsomely fitted tea-basket, in the lid of which is a silver shield bearing the following inscription: "Sister Bertha Sutcliffe, from her friends, Burgher Camp, Krugersdorp, August 1902."

### To Nurses.

We invite contributions from any of our readers, and shall be glad to pay for "Notes on News from the Nursing World," or for articles describing nursing experiences, or dealing with any nursing question from an original point of view. The minimum payment for contributions is 5s., but we welcome interesting contributions of a column, or a page, in length. It may be added that notices of appointments, entertainments, presentations, and deaths are not paid for, but that we are always glad to receive them. All rejected manuscripts are returned in due course, and all payments for manuscripts used are made as early as possible after the beginning of each quarter.

### Appointments.

Birmingham and Midland Ear and Throat Hospital.—Miss Mary J. Gordon has been appointed sister. She was trained at Perth Royal Infirmary, and has since been night sister and sister of the surgical wards at the North Riding Infirmary. She has also done nursing in South Africa as a member of the Army Nursing Service Reserve.

Burton-on-Trent General Infirmary.—Miss Blanche Williams has been appointed sister. She was trained at the General Hospital, Swansea, and has since been sister at Paddington Infirmary.

Bury Union.—Miss K. Walter has been appointed staff nurse. She was trained at Sir Patrick Dun's Hospital, Dublin.

Howard de Walden Nurses' Home and Club (the Nurses' Co-operation), 35 Langham Street, London, W.—Miss Esther H. Young has been appointed matron. She was trained at Addenbrooke's Hospital, Cambridge, where she afterwards held the post of assistant matron for over four years. She was then appointed assistant matron at Guy's Hospital, and held that post for three years, and was for a year matron of the same institution. She has done private nursing and taken charge of a private nurses' institution.

Lewisham Infirmary.—Miss Nora Isabella Fairman and Miss Rosa Maud Hooker have been appointed ward sisters. Miss Fairman was trained at the General Infirmary, Leeds, and Miss Hooker at the Manchester Workhouse Infirmary, Crumpsall.

North Bickley Union Workhouse, Clayton.—Miss Clara Stott has been appointed superintendent nurse. She was trained at the Union Infirmary, Birkenhead, and has since been night superintendent at the Royal Infirmary, Sheffield, sister at St. Giles's Infirmary, London, and sister at the Union Infirmary, Birkenhead.

North Stafford Infirmary, Hartshill.—Miss Mary W. Nayne has been appointed sister. She was trained at the London Hospital, Whitechapel, where she was afterwards staff nurse. She has since been sister at the Taunton and Somerset Hospital.

Nottinghamshire Consumption Sanatorium.—Miss Mary Henderson has been appointed matron. She was trained at Chalmers Hospital, Banff. She has since been attached to the Glasgow Co-operation of Trained Nurses, and sister of the medical ward at the Royal Alexandra Infirmary, Paisley.

Royal Albert Edward Infirmary, Wigan.—Miss Barbara Powne has been appointed sister of the children's wards. She was trained at Guy's Hospital, and has since held the post of sister at the General Hospital, Northampton, the Hospital Convalescent Home, Swanley, Kent, the Royal Victoria Hospital, Belfast, and that of night sister at the Royal Hants County Hospital, Winchester.

Royal Victoria Hospital, Belfast.—Miss Jeanette Ferrier has been appointed night superintendent. She was trained at King's Cross Hospital, Dundee, and Bolton Infirmary, Lancashire. She has since held the post of sister of enteric wards at Ruchill Hospital, Glasgow.

St. Olave's Workhouse, Ladywell, S.E.—Miss Florence Kite has been appointed superintendent nurse. She was trained at Greenwich Union Infirmary, and has since been ward nurse at Camberwell Union Infirmary, assistant nurse at Eastry Union Infirmary, and, for the past two years, superintendent nurse at Canterbury Union Infirmary.

Toxteth Infirmary, Liverpool.—Miss Christina Small has been appointed night superintendent. She was trained and has since been charge nurse of the male medical wards at the Toxteth Infirmary.

### Everybody's Opinion.

[Correspondence on all subjects is invited, but we cannot in any way be responsible for the opinions expressed by our correspondents. No communication can be entertained if the name and address of the correspondent are not given as a guarantee of good faith, but not necessarily for publication. All correspondents should write on one side of the paper only.]

### "G. O. K."

"L. P." writes, with regard to "Query 169": "G. O. K." means "God only knows." This may seem strange to you, but it is the correct definition. I heard the expression used at an operation, when the surgeon was asked what would happen to the patient.

[The expression is certainly one that should not be used.—Editor of The Hospital.]

### NURSING IN SOUTH AFRICA.

"A Nursing Sister," who has just returned from South Africa, writes: I strongly advise nurses not to go to South Africa at present unless they have some definite employment to go to. Nothing is settled at present, and board and lodging are most expensive. Private nursing in South Africa is a very different thing from private nursing in England—unless a nurse is thoroughly accustomed to colonial life, it is much better for her to remain at home. People are far too busy re-establishing their shattered homes and fortunes to think of establishing hospitals, or even to afford the luxury of a trained nurse.

### COTTAGE NURSES IN CUMBERLAND.

"A. B.," who is not a nurse and resides in Cumberland, writes: I cordially agree with your remarks in last week's Hospital *Nursing Section* "that the towns, villages, and scattered districts of Cumberland in which cottage nurses are engaged must remain at a disadvantage with other counties where fully-trained district nurses are employed." The inhabitants of the county are also not slow to see this for themselves, and in several places these cottage nurses are not employed, and yet in one instance which has come under my notice some members of a branch of the Cumberland Nursing Association have done their level best to try to drive an experienced nurse away for the sake of keeping a cottage nurse whom three-quarters of the inhabitants will not employ nor subscribe to, having by their own experience discovered the advantages of a fully-trained district nurse. Is such conduct usual?

### TAUNTON INFECTIOUS HOSPITAL.

"An Anxious One" writes: Will you allow me in the public interest to relate my experience? I, a private nurse, was sent off to the Infectious Hospital at Taunton. I got there a little after 9 in the evening, and was told by the woman in charge that I should have to do the work of three blocks with diphtheria and scarlet-fever cases. Not knowing where anything was kept, never having been to the place before, I did all I could under the circumstances, and was told the next morning I could go. But why isolate patients if nurses are sent off at a moment's notice without being properly disinfected?

[We do not, of course, know why the matron, who is not politely described by you as "the woman in charge," dispensed with your services so suddenly, but even a nurse sent off at a moment's notice from an isolation hospital shou[illegible] be allowed, and in fact compelled, to go through the proc[illegible] of disinfection.—Editor of The Hospital.]

### THE PERILS OF CARELESSNESS.

"Johanna Martin" writes: I should like to put before your readers a very sad case, the result of a piece of carelessness. We hear so often nowadays of the great care needed in ascertaining the drug in a bottle before administering the dose—in fact, we are often told to make it a habit to look twice at the label to make quite sure we have read it aright. A relative of mine, who was a trained nurse in America, went to stay for a few days in her old training-school. She was perfectly healthy, and in the best of spirits. On the night of her arrival she went upstairs to bed, and when the light was out evidently remembered that she had omitted to take some medicine. She felt along the washhandstand, and took hold of a bottle containing a strong solution of carbolic acid. Without pausing to think, she drank straight out of it. The strange part is that her sense of smell did not arrest her hand in time, but it was too late, her shriek of agony roused the household; help came almost immediately, but she was then already unconscious, and died a few hours later. When I realise the terrible suffering on her part and the great grief of her friends, and see how easily such a mistake could have been avoided, I feel compelled to speak to my fellow nurses, urging them to be careful, and to warn others to be careful, in the small things which, if neglected, can lead to such tragic consequences.

### RUPTURE OF PERINEUM.

"L.O.S." writes: Your article, reprinted, on Dr. Lyon's speech on the above subject caught my eye, and as I have now been engaged in district midwifery for ten years I feel I should like to give you my experience. Out of 1,664 cases I only had seven incomplete ruptured perineum needing stitching, and it is really a very rare occurrence. We, of course, are very careful to "foment" well at the end of the first stage in all cases of prima-para; I also lubricate freely with corrosive glycerine. My practice is to keep the occiput back during the final pains, by pressure, and I also get flexion by pressing the chin through the posterior vaginal wall. I find by letting the head immerge between the pains I can manage better to push it up off the perineum, and I also take away any pulley the patient may have used, and ask her to cry out and not to bear down. In multiparæ my experience has been that they have sustained no injury in past labours except in a very few cases, and this may partly be due to our patients being the same we have attended all through, as we get them again and again. I feel tempted to send you this account, as I think after ten years' experience I am a fair judge of midwifery, and perhaps some other nurse will be glad to know how we have been so fortunat[illegible] in our large practice.

### "PAYING PATIENTS AT LICHFIELD."

"C. K. M." writes: In The Hospital, of Sept. 13th, occur the following words:—"But there are many objections to the plan . . . of using the money earned by nurses by attending upon paying patients to help in supporting the charity of district work." Will you kindly let me know what the objections are? I can see some for myself, but I should be glad to know your views. I am hon. sec. of an association, with two nurses, who attend the really poor without charge, and we are thinking of engaging a third who would attend people who are not really very poor, such as clerks, but who could not afford a private nurse, a[illegible] charging them, say, 6d. per visit. Probably this nurse wo[illegible] not earn enough to pay for herself, but if she did, and more, would you consider it objectionable to place the surplus to the funds of the association? Our association is not affiliated to the Queen's Jubilee Institute for Nurses.

[As your Association is not affiliated to the Queen's Jubilee Institute you are not, of course, under an obligation to observe the salutary rules to which the branches are expected to conform. But, speaking generally, it is a wise policy to keep nursing the sick poor entirely apart from nursing paying patients. An association which caters for both classes of patients is naturally under the temptation, if at all pressed, to give the paying patient the preference; and it is also a vicious principle that the money earned by a nurse for attending on people who can afford to pay for her services should go to swell the funds of a charity ostensibly maintained by voluntary contributions. If the public get it into their heads that the fees of paying patients can be made to defray the cost of nursing the sick poor, they would have a legitimate excuse for withholding help to the charity of district work.—Editor of The Hospital.]

### Examination Questions for Nurses.

### OPENING OF THE AUTUMN SESSION.

Nurses are now returning from their holidays and settling in again for a year's work, bringing with them a fresh store of health and energy wherewith to face the difficulties which beset every path to a more or less degree.

I hope that our competing nurses are determined to bring to our examination-work greater care and thought, so that the improvement certainly shown during the last year or two may be continued and aggmented. Nothing stands still. Those of you who have been candidates before will write either better or worse, according to whether you put your whole mind into what you are doing, or whether you give it only a divided attention.

There are several points which I should like you to notice.

First, then, never seek to confuse the work of the doctor and the nurse; a good nurse seeks in all things to carry out the orders of the doctor in attendance on the case, and never attempts to invade his sphere with remedies and treatment of her own devising. It is extremely dangerous and improper to do so, and is besides the sign of a conceited and ill-trained woman. But common sense must be used: in the case where medical attendance can be only scanty, as when severe epidemics are raging, or in outlying districts where the doctor's visits cannot be frequent, it sometimes happens that a nurse must act on her own initiative. Let her be very cautious in doing so, and immediately explain to the medical man on his next visit what she has done, why she did it, and inquire if it meets with his approval.

I feel that it is necessary to refer to this subject because, from the nature of some of the answers received, one would imagine that the nurse believed herself to be in full charge of the patient, both for medical and surgical, as well as for nursing, treatment.

Secondly, let me beg you to refrain from "padding," and the use of long and learned terms, alas! too often wrongly spelt. Doctors greatly prefer simplicity of language; comprehension of the nature and course of the disease is needed, not a superficial acquaintance with the professional terms for its symptoms.

Thirdly, please attend to the rules; they are usually printed at the same time as the question, but, if not, the exertion of looking them up in the previous month should not be too much.

The answer must be written continuously and simply: we have no room for divisions and headings. Papers without names and addresses on the same sheet are at once discarded. Letters to the editor and examiner on the subject of the competition are not permissible.

### Question for October.

State what arrangements you would make for supporting a patient with the least possible fatigue during treatment which consists of immersion in a bath for many hours at a time?

### Rules.

The competition is open to all. Answers must not exceed 500 words, and be written on one side of the paper only. The pseudonym, as well as the proper name and address, must be written on the *same* paper, and *not on a separate sheet.* Papers may be sent in for fifteen days only from the day of the publication of the question. All illustrations strictly prohibited. Failure to comply with these rules will disqualify the candidate for competition. Prizes will be awarded for the two best answers. Papers to be sent to "The Editor," with "Examination" written on the left-hand corner of the envelope.

N.B.—The decision of the examiners is final, and no correspondence on the subject can be entertained.

In addition to two prizes honourable mention cards will be awarded to those who have sent in exceptionally good papers.

### For Reading to the Sick.

### THE BETTER LAND.

I.

Thrice blessed Land of Heavenly gladness!
  Where life is Life in endless flow;
Where undisturbed by fear or sadness
  Greatness and Peace their sweets bestow;
Where Health is pure, unchecked by sickness,
  And "laid up" Treasure never fails;
Where no more Death betrays its weakness,
  Nor Time its fleeting course bewails;
Where Happiness unyoked from sorrow
  Fills up its ample store with Love;
And face to Face, no more we borrow
  Figures to shadow God above.

*T. Skinner.*

Heaven will not be a lonely place, that we should not there have any beside God to love. Love of our brethren increases, it does not shut out, the love of God. The more we love rightly, God or man, the more power we have to love both. There all will love all. There we shall love all in God, and God in all. We shall in the love of others love God the more, because it is God whom we shall love in them. They will not be separated from God, that we could love them apart from God. God will dwell in all there; all will be transparent with His glory and His love.

. . . . .

*There*, in that abode of love, shall no special holy love be lost. God has not formed us, yea, bidden us, in this our nursery for the heavenly life, to love one another, in all our several relations, that all this, after this life, should cease. He has not bound us in those varied sweet bands of love, fathers, mothers, children, brothers, sisters, husbands, wives, friends, or those wider circles through which love radiates here, that the love which is from Himself, and which He has made part of the undying soul, shall die. We could not think, as to the very Human Nature of our Lord, that in the full glory of God He does not love still, with that same special love with which on earth He loved the disciple whom He loved.—*Pusey.*

II.

There too, we know as we are known,
  The heights of Love Divine we scan,
And see the Light from out the Throne
  Enlightening ere the worlds began;
The unfailing Food of Life is there,
  Beholding Whom our Souls are fed;
Beholding, yet with longing care
  Still to behold—still upward led.

All quickening there, with beauteous Ray
  The Sun of Righteousness is set,
Illuming all the golden Way
  Where Citizens of Heaven are met.

*T. Skinner*

In the positive joy of the great future each faculty will have its own peculiar and full happiness. "God will be the fulness of light to the reason; the fulness of peace to the will; the fulness of consolation to the memory."

"The Truth of God will satisfy the understanding; the Love of God will satisfy the will; the Providence of God will satisfy the memory."

We can understand now something of those deep words, "In Thy Presence is the fulness of joy"; or those others, "When I wake up after Thy likeness, I shall be satisfied with it."

*St. Bernard.*

### Novelties for Nurses.

BY OUR SHOPPING CORRESPONDENT.

### MESSRS. EGERTON BURNETT'S MATERIALS.

(MESSRS. EGERTON BURNETT, LTD., ROYAL SERGE WAREHOUSE, WELLINGTON, SOMERSET.)

AGAIN I have received a boxful of patterns from this noted Somersetshire firm, and I should like to draw special attention to the cloakings, ginghams, and brown holland approved by the General Superintendent for Queen's Nurses. The cloakings are dyed with a fast dye, and thoroughly shrunk and waterproofed, and Messrs. Egerton Burnett tell me that they have been much gratified by the expressions of satisfaction and commendation which they have received from superintendents and Queen's Nurses in all parts of the country. There are five qualities of material—summer, winter, intermediate, gingham, and holland—and the cloaks are made with or without a hood. The price for cloaks is from 29s. (without a hood) and 34s. (with hood), while 35s. 6d. is the highest price in the list. Gingham is from 6½d. per yard, and holland 1s. 1½d., both 27 inches wide. The cost of carriage is paid on orders of £1 and upwards in value, and there is a special discount of 5 per cent. if a whole piece of gingham or brown holland is taken. The length would be about 50 yards.

Another feature of special interest is that this firm supplies Queen's Nurses' cycling costumes in "regulation style" in navy blue serges; the prices are from 25s. 9d. to £3 10s., according to the quality of the material. The pattern shown me was a skirt which buttoned down the front, giving the appearance of a cloak, and over this was a neat tailor-made coat, three-quarter length and single-breasted, the whole surmounted by the well-known bonnet with blue velvet bow in front. All these cloaks and cycling costumes are tailor-made on the premises, and in ordering it should be stated whether the wearer is a Queen's Nurse. The box also contains a large selection of materials for indoor and outdoor ordinary dress, warm zibelines, serges and hopsacks for winter wear (the Hebrides is a particularly nice hopsack), tweeds, and coatings. Of the last, the one I like best is the Connaught coating, in grey, a colour that does not show the dust of the roads in cycling: it is 50 inches wide, and the price is 4s. 6d. a yard. The Ben Nevis is a very good tweed, and there is nothing like tweed for real hard wear. Lastly, I must mention the shrinknaught flannels for blouses, etc. The colours are pretty, and the moderate price (*1s. 7½d.*) is in itself a recommendation. They are excellent for dressing gowns and jackets, underwear, etc., while for cycling or hockey blouses it would be difficult to improve on them.

A useful thing to know is that one can get bundles of remnants from Messrs. Egerton Burnett, which are most convenient for presents if not for one's own use. I have had one (the price was 7s. 6d.) which contained material enough for two skirts; and although one cannot reckon on this, one is certain to get good quality, and a short length might perhaps be made up into a child's frock for "our Christmas distribution." There is an element of excitement in sending for a bundle when you do not know what you will get: it is like the dip in a bran-tub, or the lucky packet of our childhood; and, as I said, you are sure to get something good.

### DOMEN BELT-CORSET.

(DOMEN BELTS CO., 456 (FIRST FLOOR) CHARING CROSS, LONDON, W.C.

A SPECIALITY of the Domen Belts Company is the straight-fronted belt-corset, and to many nurses this form of stays will be welcome. It is comfortable to wear, and insures a good figure without the evils of tight-lacing, of which no nurse, or indeed any woman who knows anything of anatomy, should be guilty. In cases where further support than that afforded by an ordinary hygienic straight-fronted corset is required, this belt-corset will prove of great service, since it combines the advantages of both belt and corset. The belt is made to buckle over, crossing at the back, and as the elastic yields, it can be tightened into the next hole. The belt prevents the side bones of the corset from standing out, as they tend to do in ordinary corsets after they have been worn for some little time. The price is 19s. 6d., and only the waist measurement is required in sending an order, but it should be stated whether the long-waisted or medium short-waisted style be required.

### The Nurses' Bookshelf.

WOMAN'S BEST WORK AND LATENT CAPABILITIES. By F. Y. GANT, F.R.C.S., Consulting Surgeon to the Royal Free Hospital. (New Edition. 1 vol. Elliot Stock. 2s. 6d.)

THIS little book, we are told in the preface, is a new edition of the sequel to "Perfect Womanhood." It is re-published in an enlarged form, and contains sketches of the hospital nurse, school-teacher, deaconess, lady medical missioner, medical women. It should be interesting to those readers to whom it is specially addressed.

OPHTHALMIC NURSING. By SYDNEY STEPHENSON, M.B, C.M., F.R.C.S.E. Second edition, with 62 illustrations. (London: The Scientific Press. 1902. Price 3s. 6d. net.)

SPECIALISM creeps into nursing as well as into medicine, and so far as specialism means added skill, something beyond that which is possessed by the generality, specialism is to be welcomed. A very slight perusal of this book will show the nurse whose training has been chiefly obtained in the general wards how much there is in ophthalmic work that is fresh and different from what is seen elsewhere, just as a very short experience in almost any of the special departments of a hospital will make her feel how imperfect is a training which is "general" only; and it is because of the peculiar degree to which ophthalmic work has been specialised that nurses will benefit from the use of a manual like the one before us. After giving a good general description of the eye, the author turns to that keynote of all modern surgery, the germ theory of disease. Then, as is natural, in view of the large part played by contagion in the production of many diseases of the eye, we have a chapter on the contagious diseases of this organ, giving an account also of the special precautions which have to be adopted to prevent their spread. Ophthalmic remedies are then treated of, and the special methods adopted for their application to the eye. Then we come to the operations, the nature and to some extent the objects of which nurses must be familiar with, if they are to give intelligent and useful assistance to the operating surgeon. All these things are useful, but what is told about the nursing of the various operations, and of the various diseases of the eye, may be regarded as essential, dealing as it does with matters about which a nurse who has to do with ophthalmic patients is sure to require information. Finally there is a very useful appendix, giving illustrations of the various instruments used—a matter of considerable importance, for a surgeon who is in the midst of an operation or even a dressing, cannot take his eye from his patient to look for an instrument, so the nurse must know what they are called. At the end of the book there is a glossary of terms, which will be found useful to some. The book is evidently the outcome of much experience, and ought to prove very acceptable to nurses.

### Echoes from the Outside World.

### The King's Visit to South London.

Replying to a letter from the Mayor of Southwark, Lord Knollys says that he is not at present in a position to determine the character and length of the procession required to accompany the King and Queen on the occasion of the Royal progress in South London. Lord Knollys has also sent a letter to the Mayor of Battersea, who had expressed a desire that His Majesty should visit that borough at an early date, in order to distribute a few of the 30,000 Coronation medals to school children, in which he says that if the King were to distribute the medals it would open the door for other boroughs to make the same request. "The King, moreover," added Lord Knollys, "is very unwilling, except in very special cases, to perform public ceremonials during the winter months."

### The late Queen of the Belgians.

There was a solemn memorial service in Brussels Cathedral on Thursday, last week, for the late Queen of the Belgians. The whole of the sacred edifice was draped in black, an immense canopy was suspended from the roof, extending over the high altar to the right of the Royal throne, whose velvet and gold decorations were completely covered with crape. The catafalque was erected under the transept, and was surmounted by the Royal mantle and crown. It was surrounded by four rows of wax tapers bearing the Royal arms of Belgium and Hungary. The procession, which was headed by the clergy, bishops, and cardinal archbishop, included the king, members of the Royal Family, high State officials, representatives of foreign nations, the civil and military authorities, the senators, the deputies, and the Belgian ministers. During the service the choir sang a requiem mass, and on leaving the cathedral the King with Princess Clementine led the procession. A large crowd of people, who behaved admirably, collected near the approaches and remained until the mourners left the building.

### The Graves on the Veld.

An order has been issued to the South African Constabulary by Major-General Baden-Powell with reference to the preservation of the graves of those who have fallen in the war. The General says: "I want N.C.O.'s and men in out-stations to do all that they can, without letting it interfere with other duties, to help in the identification and preservation of the graves. Very many of these lie scattered over the veld, in out-of-the-way places, where none but constabulary will be likely to go, and it is only due to the dead and to their relatives that we should do what we can to keep their graves and monuments in good order. And I hope that in caring for the resting-places of our own folk, we shall equally regard those of our former enemies, the Boers, who fell fighting bravely for their cause and equally deserve our respect."

### The Boer Appeals.

Last week a document was issued entitled, "Appeal of the Boer Generals to the Civilised World," signed by General Botha, De Wet, and Delarey, in the course of which they stated that, as they had not succeeded up to the present in inducing the British Government to grant further assistance to "aid people in their indescribable distress," it only remained for them to address themselves to the peoples of Europe and America. Concurrently with the issue of this appeal is was announced that Mr. Henry Phipps, of the Carnegie Steel Trust, had contributed a hundred thousand dollars to the Boer fund, with reference to which Mr Chamberlain—in reply to a letter sent him by Mr. Arnold White—says that "the fund would, of course, appeal more strongly to English sympathy if it were for the joint object of assisting, without distinction of race or politics, all widows and orphans who have suffered in consequence of the war." Meanwhile, it appears that the local Boer war-relief funds amount to a total of £62,778, of which £17,975 has been invested at 4 per cent. interest.

### Cyclone in Sicily.

A cyclone which took place in Sicily on Friday last week, involved the loss of 200 lives, and damage to the extent of several million lire. At Scicli the torrent swept away a number of houses with their inhabitants, and the lower portion of Modica has been partially destroyed. The two mountain torrents of San Francesco and Santa Maria, which course through the town, were suddenly flooded and carried far into the country streams of mud and large stones, at the same time invading houses and shops and washing away human beings, furniture, provisions, and animals. A German steamer foundered at the entrance to the harbour of Catania after a hard struggle with the waves. On Friday night Stromboli was in full eruption. Lava ran down the mountain sides to the sea, whilst large boulders were hurled from the crater of the volcano into the air to a great height and then fell into the sea fully four kilometres from the shore. Around Mount Etna, which has been showing signs of activity, a thick column of steam ascending from the scene of the eruption of 1892, the vineyards have been partially destroyed, and on Sunday the storm was so violent that a goods-train was blown off the rails between the stations of Vasto and San Salvo. Every effort is being made to send pecuniary help to the relief of the sufferers, King Victor Emmanuel having headed the list with 50,000 francs. He keenly feels the misery of his subjects, and would have gone South to assist by his presence, but the doctors are most anxious that he should not do so, because any anxiety might act badly upon the health of the Queen.

### Death of M. Zola.

On Monday morning M. Zola was found asphyxiated in the bedroom of his house in the Rue de Bruxelles, Paris. He was discovered by his servant at nine o'clock in the morning lying upon the floor of his room quite dead, whilst Madame Zola was in bed, gasping for breath. Medical men were at once sent for, and after a short time the wife was restored to consciousness, but all efforts to induce respiration in M. Zola were unavailing. As far as can be at present ascertained, owing to the defective state of the chimney, fumes from the fire which had been lighted to take the chill off the room had escaped into the apartment, and caused asphyxiation. According to directions given by Madame Zola overnight, the workmen arrived to repair the defect in the chimney just about the time when M. Zola breathed his last. Most of the French papers panegyrise the talents of the author of "L'Assommoir," and *La Patrie* says he must be hailed "as one of the greatest novelists in French literature," but there still remains in some quarters a feeling of bitterness against him for the courageous part which he played in the Dreyfus case, and it has been said that at the time of the trial he was the "best-hated man in France." The sale of his books produced for him an income of at least £4,000 a year. His married life was a particularly happy one.

### Notes and Queries.

**The Editor is always willing to answer in this column, without any fee, all reasonable questions, as soon as possible.**

**But the following rules must be carefully observed:—**

**1. Every communication must be accompanied by the name and address of the writer.**

**2. The question must always bear upon nursing, directly or indirectly.**

**If an answer is required by letter a fee of half-a-crown must be enclosed with the note containing the inquiry, and we cannot undertake to forward letters addressed to correspondents making inquiries. It is therefore requested that our readers will not enclose either a stamp or a stamped envelope.**

*Home.*

(1) Will you kindly tell me if there is any home for inebriates where a lady almost without means could be received?—*M. E. W.*

The Secretary, the National British Women's Temperance Association, 47 Victoria Street, London, S.W., would be able to advise you.

I should like to know as soon as you can tell me of a home near London where an old woman could be received on the payment of 7s. a week. She is in good general health, but mentally she is rather weak.—*Matron.*

If the poor woman is a Roman Catholic the Little Sisters of the Poor, Portobello Road, W., or the Sisters of Nazareth, Hammersmith, might take charge of her. The difficulty, of course, is that she requires personal attention. Such patients are best taken care of in the workhouse infirmary.

Will you tell me the addresses of any homes where a poor woman's idiot-child could be received without payment?—*Sister D.*

You had better apply to the Secretary of the Scottish National Institution for the Education of Imbecile Children, Larbert, Stirlingshire.

Can you tell me of a charitable institution which would take a poor woman, weak in body and mind, free of charge? She has a great longing to be in the country amongst poultry, and could pay a small sum weekly.—*Miss W.*

See our reply to *Matron.* Perhaps some of our readers know of a suitable cottage-home where she would be kindly treated.

Can you tell me where an old invalid lady of 83 could be taken care of? She is bedfast but gives little trouble, is cheerful and suffers no pain. Her children could contribute about 10s. or 12s. 6d. a week to her maintenance.—*M. R. C. S.*

Possibly the Hon. Secretary, Woodside Home, Whetstone, N., could make some arrangement with regard to this case.

Can you tell me of a home where a poor woman aged 68 could be sent for a small sum weekly? She suffers from slight paralysis and aneurism of the heart. Of course I know that there are plenty of places where people of means can be taken care of, but I want to hear of some place suitable for poor people who can only pay a very small amount.—*E. S.*

We are constantly asked the same question you have put to us. But when it is taken into consideration that the ratepayers provide everything that is necessary for such cases in the workhouse infirmaries, and that if there is room patients who can only pay small amounts are admitted, it seems a pity to send them to cheap homes where, unless liberally supported by private charity, they may not obtain the comforts, even luxuries, provided for them under the Poor Law. See reply to *Matron.*

*Nurse C.* would be glad to know where she could get information regarding a society which would help an old lady of 72. She has been a widow for 25 years. Is there not a poor ladies' benevolent society?

The National Benevolent Institution, 65 Southampton Row, W.C., and the United Kingdom Beneficent Association, 7 Arundel Street, Strand, W.C., both assist persons of the upper and middle classes in reduced circumstances. Apply to the Secretaries.

Will you please tell me of a nursing home in Liverpool where an infirm elderly woman of small means could be taken care of?—*W. G.*

There is a Home for Widows at 108 Huskisson Street; and a Home for Single Women at 38 Upper Pett Street, Liverpool, but we have not any particulars concerning them.

Is there a home at Bournemouth where a convalescent patient could go for two or three weeks, paying about 25s. weekly?—*R. A. B.*

The Hahnemann Convalescent Home and Dispensaries, West Cliff, Bournemouth, receives convalescents for £1 1s. a week.

Can you tell me of a home where a very anæmic girl can be received for a slight payment? She is quite unprovided for, and her friends are not in a position to do much for her. She could help in light work. The north of England preferred.—*Nurse Dora.*

You do not mention the social position of the girl. Consult a medical man as to the best place for her to go in order to recover her health.

Can you kindly tell me of any institution which will receive a deaf and dumb boy of eight? His mother is a widow, and very poor.—*Miss C.*

The Royal Asylum for the Deaf and Dumb Poor, office, 93 Cannon Street, London, E.C., seems the most suitable charity for this case.

*Radiant Light Cure.*

(2) I would be obliged if you could tell me of any hospital or institution where I could receive the radiant light and heat treatment. I am crippled with rheumatoid arthritis, but have no means by which to pay for it.—*Grateful.*

You might apply to the Dowsing Radiant Heat Company, 24 Budge Row, Cannon Street.

*Nurses' Club.*

(3) Will you kindly tell me if there is a nurses' club in London?—*Nurse D.*

The Trained Nurses' Club, 12 Buckingham Street, Strand, W.C.

*Employment.*

(4) Can you kindly tell me to whom I should apply for an appointment on the staff of a Government district hospital, or for a Lady Dufferin's Fund hospital, or for one under the Up-country Nursing Association in India?—*F. B.* and *Nurse B.*

Apply the Secretary of the Colonial Nursing Association, Imperia Institute, S.W., for Government appointments abroad; to the Secretary, the National Association for Supplying Female Medical Aid to the Women of India, United Kingdom Branch, 9 Clarence Place, Belfast, for information as to the appointments in the hospitals supported by Lady Dufferin's Fund; and to Mrs. Sheppard, 10 Chester Place, Regent's Park, N.W., for particulars of the rules of employment and of the work of the Up-country Nursing Association.

I should be glad if you could tell me how I could obtain the post of matron in a cottage hospital or small home on the South Coast, in which my invalid daughter could also be received. She suffers from chronic rheumatism and consumption. I am a good housekeeper.—*M. M. M.*

You can only obtain such an appointment by advertising.

I am a fully qualified nurse endeavouring to join the Nurses' Co-operation. Should I fail, could you tell me of any other association conducted on similar principles, which would help me? I speak both French and German, and would like a remunerative appointment abroad. Is there any international association?—*K. T. P.*

There are a number of nurses' associations, but none exactly on the same lines as the Nurses' Co-operation. Several of them advertise in our columns. As you have applied to the Nurses' Co-operation, the secretary will have seen your testimonials, and she would probably be glad to give you advice if she cannot avail herself of your services.

I should be very glad if you will kindly tell for what post I could apply and how to do so? I am married and a trained nurse. I have held several appointments under the Local Government Board, and was appointed matron of a small hospital before my marriage.—*T. E. T.*

You could not do better than apply for an appointment under the Local Government Board. In some Poor Law institutions the matron is permitted to be married. Advertise in the Poor Law journals.

*Abroad.*

(5) Will you kindly tell me the name and address of a nursing home either in Johannesburg or Queenstown, S.A.?—*A. A. C.*

Apply to the Superintendent Nurse, the Co-operative Residential Club, Johannesburg, or to the Lady Superintendent, the Victoria Nurses' Institution, Capetown, Cape Colony.

**Standard Books of Reference.**

**"The Nursing Profession: How and Where to Train." 2s. net; post free 2s. 4d.**

**"Burdett's Official Nursing Directory." 3s. net; post free, 3s. 4d.**

**"Burdett's Hospitals and Charities." 5s.**

**"Hospital Expenditure: The Commissariat." 2s. 6d.**

**"The Nurses' Dictionary of Medical Terms." Cloth, 2s.; leather, 2s. 6d. net.; post free, 2s. 8d.**

**"Burdett's Series of Nursing Text-Books." 1s. each.**

**"A Handbook for Nurses." (Illustrated). 5s.**

**"The Physiological Feeding of Infants." 1s.**

**"The Physiological Nursery Chart." 1s.; post free, 1s. 3d.**

**All these are published by the Scientific Press, Ltd., and may be obtained through any bookseller or direct from the publishers, 28 and 29 Southampton Street, London, W.C.**

### Travel Notes.

**By Our Travelling Correspondent.**

### CXI.—"THE VERY VALLEY OF THE SHADOW OF DEATH."

These words occur in Macaulay's magnificent description of this tragic spot. It reads like the finest romance, but then is not reality generally far more extraordinary and dramatic than the wildest fiction? The spot seems created for some weird and awful tragedy, the splendour of its loneliness and isolation cannot be surpassed; and if you are fortunate enough to visit it in early June or late October, when tourists are absent, it leaves a more impressive effect on your mind.

### First Sight of Glencoe.

Glencoe looks at its best from N. Ballachulish. The two mountains, which appear to close the lake entirely, called Sgor na Ciche and Garbh Bheinn are very grand, and catch the reflection of the setting sun long after the valley is in shadow. To go up the Pass you must cross the ferry to Ballachulish proper and book seats in Cameron's coach; this only costs 5s. 6d., but it is naturally much pleasanter to have a carriage to yourself, and if you are a numerous party it is worth while to do so, especially as the coaches heavily loaded cannot go so far as is desirable up the glen. I have seen the place under two aspects, in brilliant sunshine, when, even desolate though it is, there is perforce much of beauty in the delicately tinted mountains and scanty herbage at their feet, and again in a gloomy, fiercely windy day of storm and cloud in chill October. This latter view seemed more in accordance with the grim and ghastly story of treachery and murder which have clothed the spot with interest for more than 200 years. The facts are briefly as follows:—

### The Massacre of the MacIans.

In 1691 William III.'s Government issued a proclamation which it was hoped would quiet the disaffected Highlands and bring all under one flag. This proclamation called upon all Highland heads of clans to take the oath of allegiance to William and Mary by December 31st in that year, or failing to do so they would be subject to martial law. All had complied with the edict except MacIan, head of the MacDonalds, who, apparently by an oversight, had allowed the allotted time to almost expire before going to Fort William to take the oath. Arrived there he was told that there was no civil officer before whom such oaths could be administered, but Colonel Hill, the governor of the fort, provided him with a kind of written protection and told him to hasten down to Inverary. The weather was severe, the snow deep on the hills, and the chieftain aged, so that the journey occupied some time and the oath was not taken until January 6th.

It has always appeared that Viscount Stair, of the Privy Council, was largely to blame for what followed, but it is difficult to arrive at the truth, and in spite of Macaulay's impassioned attempt to whitewash William's conduct, it must ever remain a hideous blot upon that monarch's life and reign.

On February 1st a body of over 100 soldiers appeared in the neighbourhood of the glen and stealthily closed all exits, even taking precautions that no boat should remain at the south ferry to assist hapless fugitives.

The soldiers were commanded by Campbell of Glenlyon, who, being the uncle of young MacIan's wife, it seemed not unnatural should be billetted in the chief's house; the excuse for their presence being that the barracks were over full at Fort William, and it was desirable to make a show of strength in the district. Accordingly the three officers were accommodated in the chief's house and the soldiers distributed in the little hamlet. The ruined houses are still seen in the same condition.

Things went quietly for a fortnight, though tradition says that a seer warned MacIan of impending danger; then the crash came. A junior officer called late at night and desired to speak with the chief, who, before he could dress, was shot dead, whilst his wife was stripped and the rings torn from her fingers.

This was the signal for a general massacre, and it was through darkness and other causes that any male member of the clan escaped. Sixty met their deaths, but about 150, chiefly women and children, escaped into the caves of the mountains, many of them to perish by cold and starvation.

The following ghastly and succinct account occurs in the evidence in the Parliamentary report of 1695: "Where Glenlyon was quartered, the soldiers took other nine men and did bind them hand and foot and killed them one by one with shot; and when Glenlyon inclined to save a young man of about twenty years of age, one Captain Drummond came and asked how he came to be saved, in respect of the orders that were given, and shot him dead; and another young boy of about thirteen ran to Glenlyon to be saved. He was likewise shot dead; and in the same town there was a woman, and a boy about four or five years of age killed. And at Achnacon there was also a child missed and nothing found of him but the hand. There were likewise several killed at other places, whereof one was an old man about eighty. And all this, the deponents say, they affirm because they heard the shot, saw the dead bodies, and had an account from the women that were left." Near the entrance of the glen a memorial is erected to the memory of the murdered MacIans by a Mrs. Burns, who was their direct descendant. About a mile farther up is a huge boulder from which it is always maintained that the shot was fired which was the signal for the slaughter. If you are *very* good walkers (not otherwise) there is a beautiful round to be made by going past the "study," as it is called, where the finest view of the Pass is obtained, on to the King's House, a small hostelry, and so across by the Devil's Staircase to the head of Loch Leven and home on the other side of the loch. It would make a round of quite 26 miles, and even a good pedestrian can only manage it by going on one of the coaches as far as he can and walking the rest; by this means he cuts off 14 miles. The turn for the Devil's Staircase occurs quite two miles on the Glen side of King's House.

### TRAVEL NOTES AND QUERIES.

Living in France for the Winter (Hedwig).—You need not apologise for the length of your letter; it makes it far easier for me to help you when you are explicit. Life in France is somewhat different to that in Germany. The French are not inclined to wander so much, nor do they affect pensions, with the result that I feel you will meet too many of your own country-people in such houses, which will defeat your object of perfecting yourself in the language. Tours is a nice bright little town, and has the reputation of "perfect French." Of pensions there I know nothing, though I believe there are several. At the moment I cannot think of anything that really meets your requirements, except in French Switzerland. In the neighbourhood of the Lake of Geneva I believe I could find just what you want. Let me hear again. I could give you a long list of pensions in France, but they are all frequented by English. How would you like a family? I know of two, one in Caen and one in Pau. You might find it a little dull, but it is the way to learn the language. Pau is a lovely spot, and bright for a person alone. Tell me what you think of these ideas, and also how Switzerland would suit. A correspondent of mine spent four months of last winter above Lausanne and enjoyed herself greatly.

## Figures and Tables

**Fig. 61. f1:**
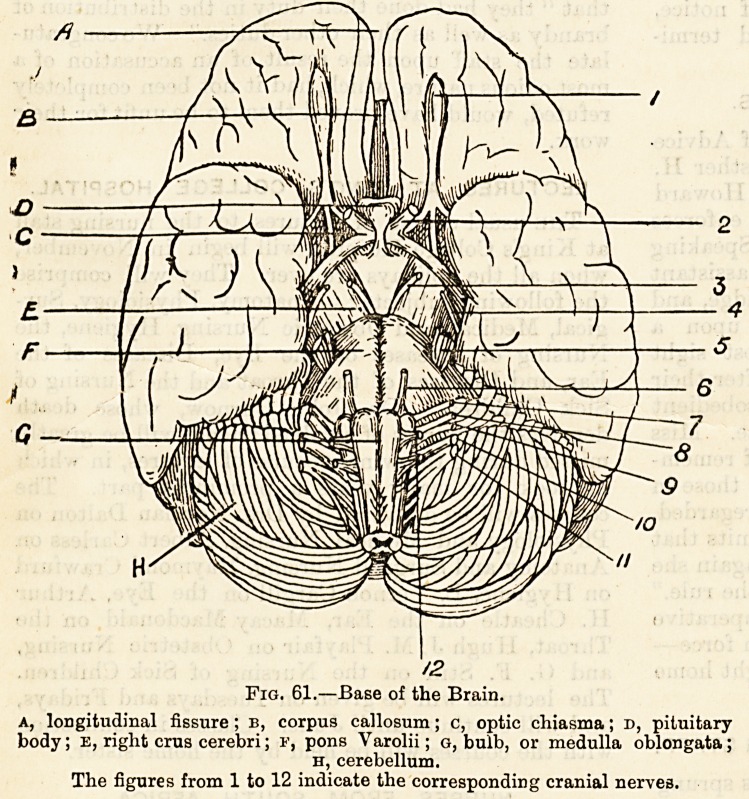


**Figure f2:**
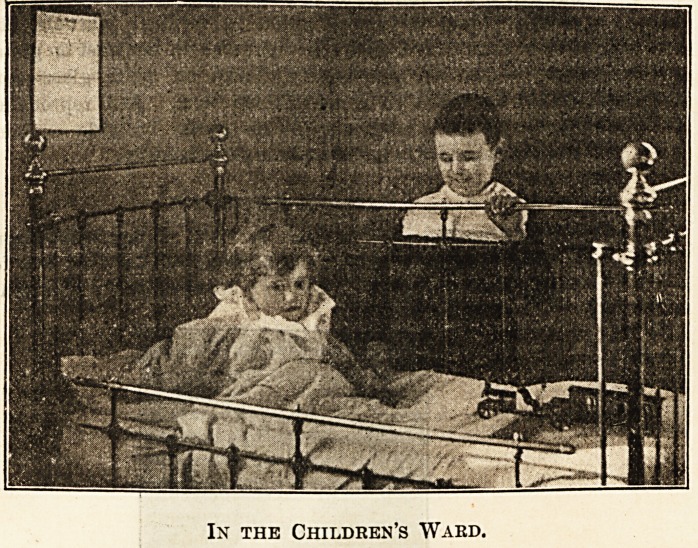


**Figure f3:**
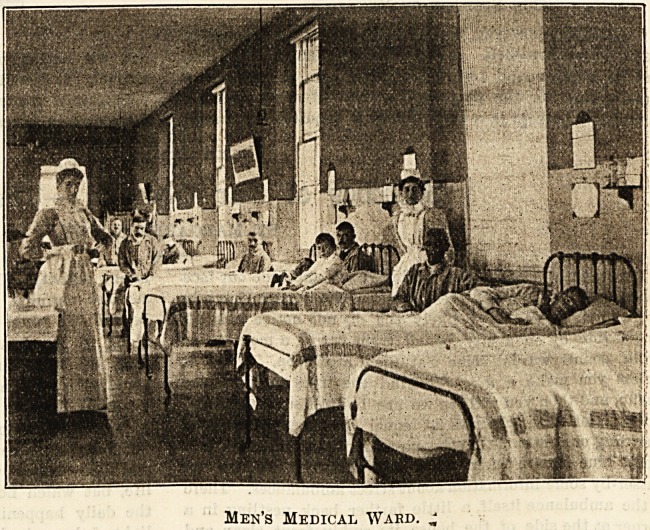


**Figure f4:**
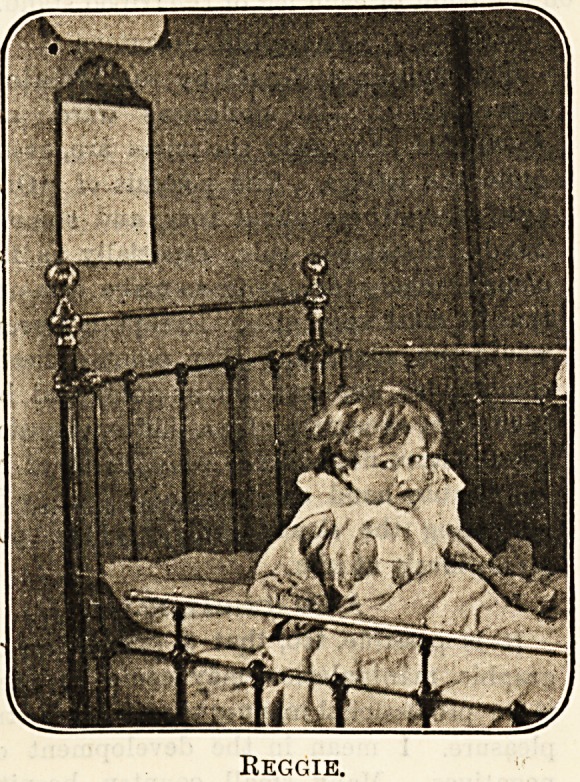


**Figure f5:**